# Physiological and clinical effects of two ultraprotective ventilation strategies in patients with veno-venous extracorporeal membrane oxygenation: the ECMOVENT study

**DOI:** 10.1186/s13613-025-01525-0

**Published:** 2025-08-01

**Authors:** Yorick Rodriguez, Alexandre Thomachot, Guillaume Deniel, Mehdi Mezidi, Louis Chauvelot, Hodane Yonis, Jean-Christophe Richard, Laurent Bitker

**Affiliations:** 1 Service de Médecine Intensive—Réanimation, Hôpital de La Croix Rousse, 103, Grande Rue de La Croix Rousse, 69004 Lyon, France; 2 Université Claude Bernard Lyon 1, Lyon, France; 3 Univ Lyon, Université Claude Bernard Lyon 1, INSA-Lyon, CNRS, INSERM, CREATIS UMR 5220, U1294, Villeurbanne, France

**Keywords:** Extracorporeal membrane oxygenation, Ultraprotective ventilation, Mechanical ventilation, Mechanical power, Ventilator-induced lung injuries

## Abstract

**Purpose:**

The optimal ventilation strategy in acute respiratory distress syndrome (ARDS) patients with veno-venous extracorporeal membrane oxygenation (VV-ECMO) remains unknown. We aimed to compare the effects of two ultra-protective ventilatory strategies applied to patients with ARDS and VV-ECMO.

**Methods:**

Our study was an observational, retrospective, single-center study with a before-and-after design. All consecutive patients treated with VV-ECMO for severe ARDS between 2016 and 2023 were included. Before 2021, patients received a quasi-apneic ventilation strategy in assist-controlled volume mode with a tidal volume (V_T_) of 1 ml.kg^−1^ predicted body weight (PBW), a respiratory rate (RR) of 5 min^−1^ and a PEEP set to keep plateau pressure (P_PLAT_) between 20 and 25 cmH_2_O. From 2021 onwards, the protocolized ventilatory strategy consisted in pressure-controlled mode with a PEEP of 14 cmH_2_O, a driving pressure (∆P) of 8 cmH_2_O and a RR of 10 min^−1^. We evaluated the impact of strategies on longitudinal respiratory mechanics and on the time to successful ECMO weaning at day-90 after VV-ECMO canulation.

**Results:**

121 patients were enrolled, with 69 receiving the VT1 strategy, and 52 the ∆P8 strategy. Over the first 7 days of ECMO, the ∆P8 strategy was associated with significantly higher ∆P and RR, lower PaCO_2_, and higher static elastic mechanical power, compared with the VT1 strategy. The day-90 survival rate was 30% with the VT1 strategy, and 42% with the ∆P8 strategy (*P* = 0.19). Time to successful VV-ECMO weaning was 7 [4–13] days in day-90 survivors, with no significant difference between groups. The adjusted subdistribution hazard ratio associated with the ∆P8 strategy was 0.99 (95% confidence interval: 0.53–1.84), as compared to the VT1 strategy (*P* > 0.9).

**Conclusions:**

In the context of our center, a ventilatory strategy targeting a PEEP of 14 cmH_2_O, a ∆P of 8 cmH_2_O and a RR of 10 min^−1^ led to the application of ∆P, RR and static elastic mechanical power and improved decarboxylation, compared to a strategy in volumetric mode with a V_T_ of 1 ml.kg^−1^ PBW and a RR of 5 min^−1^, in patients with ARDS and VV-ECMO. No significant difference on clinical outcomes was observed between both strategies.

**Supplementary Information:**

The online version contains supplementary material available at 10.1186/s13613-025-01525-0.

## Introduction

Although invasive mechanical ventilation is the cornerstone therapy in patients with acute respiratory distress syndrome (ARDS), inadequate settings may lead to *ventilator-induced lung injuries* (VILI), consisting of *barotrauma*, *volutrauma* and possibly *biotrauma* [1, 2], which may further adversely impact patient outcome [3]. Especially, the most severe ARDS patients are exposed to a significantly higher risk of VILI due to the extent of lung parenchymal abnormalities and the subsequent decrease in aerated lung volume [4]. In these patients, veno-venous extracorporeal membrane oxygenation (VV-ECMO) has the double advantage of maintaining oxygenation and decarboxylation while theoretically allowing the application of so-called ultra-protective ventilatory strategies aiming at preventing VILI [5].

To date, the optimal ventilation strategy in ARDS patients with VV-ECMO remains a subject of debate. In the EOLIA randomized controlled trial, patients randomized to the ECMO arm received a ventilation strategy which targeted high positive end-expiratory pressure (PEEP, approx. 10 cmH_2_O), a driving pressure (∆P) of 14 cmH_2_O and a respiratory rate (RR) between 10 and 30 min^−1^, which did not translate into a significant survival benefit, compared to conventional protective ventilation without VV-ECMO [5]. The international observational study LIFEGARDS reported ventilatory settings similar to the EOLIA protocol, with a resulting V_T_ of 3.7 mL.kg^−1^ PBW [6]. The extracorporeal life support organization (ELSO) recommends to maintain a P_PLAT_ < 25 cmH_2_O with a positive end-expiratory pressure (PEEP) > 10 cmH_2_O [7]. On the other hand, animal models and human studies have observed that ultra-protective ventilatory strategies aiming to reduce V_T_ and RR to a quasi-apneic state (1–2 ml.kg^−1^ PBW, RR < 10 min^−1^) decreased mechanical power (MP_RS_) and possibly *biotrauma*, but with uncertain clinical benefits, since they may increase the risk of alveolar collapse due to low V_T_ [8–10].

Some technical and physiological issues may arise from these quasi-apneic strategies, first in relation with the inability of adult circuit humidification and heating devices to perform adequate humidified ventilation when minute ventilation is significantly reduced [11], and may justify the use of pediatric circuits to prevent airway obstruction. Also, very low V_T_ ventilation may participate to the reduction of the baby lung by reducing intra-tidal recruitment, and expose patients to further reduction in lung compliance and prolonged ECMO support. Finally, even when a V_T_ of 1 ml.kg^−1^ PBW is applied in volume-controlled mode, P_PLAT_ may exceed 25 cmH_2_O despite the reduction in set PEEP, and ∆P may exceed safety thresholds in the most severe cases. Oppositely, a pressure mode strategy with a fixed inspiratory and ∆P will adjust the V_T_ to the individual’s baby lung, protecting lung units from barotrauma and overdistension.

Our hypothesis was that a pressure-controlled ventilatory mode with high PEEP and low ∆P may prevent alveolar collapse and limit VILI, compared to a quasi-apneic ventilation strategy in assist-control volume ventilation mode. The study’s objective was to compare the impact of two ultraprotective ventilatory strategies on lung respiratory mechanics, and their association with the time to successful ECMO weaning, in patients with severe ARDS.

## Methods

### Study design

Our study was an observational, retrospective, single-center cohort study with a before-and-after design, conducted in the medical intensive care unit of the Croix-Rousse academic hospital in Lyon, France. The study’s protocol was reviewed by the Hospices Civils de Lyon human ethics committee (reference number 24–5440) and was compliant with French data protection regulations. Given the non-interventional nature of the study, the committee required that all included patients be informed of the utilization of their data, without requiring their signed consent. The present report follows the STROBE recommendations for the report of observational studies [12].

### Study population

All consecutive patients admitted to the medical intensive care unit (ICU) of the Croix Rousse hospital between December 1st, 2016 and January 31st, 2024 and treated with VV-ECMO for severe ARDS (Berlin definition) were included [13]. Exclusion criteria were: age < 16 years, presence of severe chronic respiratory failure (home non-invasive ventilation or oxygen support), patient under a legal protective measure, previous inclusion in the same study during a prior ICU stay, and lack of consent for data utilization by patients or their legal representative. Patients with unilateral pneumonia were not considered eligible.

### Study groups

Over the period of inclusion, our center implemented a change in practice on June 1st, 2021 that affected the preferred ventilatory mode and settings in patients receiving VV-ECMO. In short, before this date, patients received a quasi-apneic ventilation strategy in assist-controlled volume mode with a V_T_ of 1 ml.kg^−1^ PBW and a RR of 5 min^−1^ (“before” period, the “VT1” strategy group). From June 2021 onwards, the ventilatory strategy was modified and consisted in using a controlled-pressure mode (either BIPAP or APRV) with a set PEEP of 14 cmH_2_O, a ∆P of 8 cmH_2_O and RR of 10 min^−1^ (“after” period, the “ΔP8” strategy group). These two periods (and related protocolized strategies) defined the study groups. We referred to VT1 or ΔP8 strategy when discussing the bundle of protocolized ventilatory settings. Further details regarding ventilatory strategies are given below.

### Study physiological and clinical outcomes

Physiological outcomes comprised longitudinal respiratory mechanics (set PEEP, P_PLAT_, mean airway pressure, RR, V_T_, respiratory system normalized elastance (E_L,PBW_), dynamic and static elastic MP_RS_), blood gas results, and ECMO settings, were collected on ECMO canulation day (i.e. day 1, before and after ECMO canulation and application of ventilatory strategies), day 3 and day 7 after canulation. Also, in patients also enrolled in the CT4ARDS2 study (NCT06113276), we also considered the following outcomes’ comparison between study groups: non-inflated lung mass at PEEP 5 cmH_2_O, end-expiratory aerated lung volume at PEEP 5 cmH_2_O, tidal hyperinflation, and lung recruitment between PEEP 5 and 15 cmH_2_O.

The impact of ventilatory strategies on time to successful VV-ECMO weaning at day-90 after VV-ECMO canulation (with no distinction between death and end of follow-up for censoring the data) was also assessed. Patients who died during the ECMO run were censored at time of death, and patients successfully weaned but who later died before day 90 were censored at time of weaning. Patients alive and still under VV-ECMO at day 90 were censored at day 90. Other clinical outcomes included all-cause mortality at day 90, ECMO-free days, ventilator-free days, length of ICU and hospital stay. Successful ECMO and ventilatory weaning were defined as being alive without the occurrence of a second ECMO run or re-intubation within 7 days following technique liberation, respectively. Organ support-free days were defined as the period of time without support (between the date of successful weaning and day-90) censored at day-90. Patients still under organ-support at day-90 (ECMO or mechanical ventilation, respectively) received a score of 0 day. Patients who died before or at day-90 also received a score of 0 day.

Data collection methodology and study time points are further detailed in Supplemental Methods.

### VV-ECMO eligibility, management and weaning

Patients eligible for VV-ECMO had ARDS as per the Berlin definition, and one of the following conditions: a ratio of arterial oxygen partial pressure (PaO_2_) to the fraction of inspired oxygen (FiO_2_) < 60 mmHg for 3 h or more despite neuromuscular blockade (NMB), prone positioning and ventilatory optimization; or a PaO_2_/FiO_2_ ratio < 80 mmHg during 8 h or more despite NMB, prone positioning and ventilatory optimization; or a pH < 7.15 despite an increase in VT > 8 ml.kg^−1^ PBW with a RR of 35 min^−1^ and a P_PLAT_ < 28 cmH_2_O after dead space minimization and NMB [14]. For analytic purposes, the two “refractory hypoxemia” conditions were merged, in opposition to the refractory hypercapnia condition. ECMO non-eligibility criteria are reported in Supplemental Methods.

The ECMO blood flow and the fraction of delivered oxygen (FdO_2_) were adjusted to maintain a PaO_2_ between 55 and 80 mmHg or an arterial oxygen saturation (SaO_2_) between 88 and 95%. Sweep gas flow was adjusted to maintain arterial pH between 7.25 and 7.45 and an arterial CO_2_ partial pressure (PaCO_2_) < 45 mmHg. ECMO protocolized weaning was performed daily, starting 48 h after ECMO canulation. Procedure’s details are given in Supplemental Methods.

Patient care was formalized based on international recommendations for ARDS management and the local ICU protocol [14, 15]. Details regarding sedation, proning, NMB use, steroids and therapeutic hypothermia are given in Supplemental Methods.

### Ventilatory strategies

Mechanical ventilation was performed with Evita XL (Dräger, Lubeck, Germany), Evita Infinity® V500 (Dräger, Lubeck, Germany), Puritan Bennett™ PB850 (Puritan Bennett, Overland Park, KA, USA), or Carescape R860 (GE Healthcare, Chicago, IL, USA) ventilators.

During the before period (VT1 strategy), the ICU protocol mandated that patients with VV-ECMO be ventilated with the following settings: ACV mode, V_T_ 1 ml.kg^−1^ PBW, PEEP adjusted to maintain P_PLAT_ between 20 and 25 cmH_2_O, RR of 5 min^−1^, an inspiratory to expiratory ratio (I:E) of 1:2. The FiO_2_ was set equal to the FdO_2_. Given the low V_T_ and low inspiratory flows and to allow adequate humidification and heating of medical gas by the automatic MR850 humidifier (Fisher & Paykel, Auckland, New-Zealand), these settings required the use of pediatric circuit with a heated wire.

During the after period (∆P8 strategy), the ICU protocol mandated that patients with VV-ECMO be ventilated with the following settings: BIPAP or APRV mode, set PEEP at 14 cmH_2_O, high pressure of 22 cmH_2_O (i.e. a ∆P of 8 cmH_2_O), RR of 10 min^−1^, an inspiratory time of 2 s (implying a I:E of 1:2 at RR of 10 min^−1^). These settings did not require the use of pediatric circuits (as assessed by the presence of moist in the endotracheal tube, and the absence of temperature alarm on the MR850 device). Lower pressure settings were considered in case of proven barotrauma (pneumomediastinum, pneumothorax).

The strategies were to be maintained during the whole duration of the ECMO run. Spontaneous ventilation was not used during ECMO support. The criteria defining per-protocol application of ventilation strategies are detailed in Supplemental Methods.

### Quantitative computed tomography study

The CT4ARDS2 study enrolled patients with ARDS with ECMO for less than 3 days and with an indication to perform a lung computed tomography (CT) scan [16]. The methodology of quantitative CT analysis is described in Supplemental Methods.

### Statistical analysis

Sample size computation could not be performed given the absence of available data to estimate an effect size with the compared strategies. Hence, a convenient sample was analyzed, comprising all consecutive patients fulfilling inclusion criteria since the first VV-ECMO admission in our center.

Two subpopulations were also considered for analysis. First, a per-protocol population was identified, defined as patients who successfully received ventilation with settings as mandated by the protocol on day 1 of ECMO run. Second, a pseudo-population was considered, using propensity score matching, to compare the clinical impact of ventilatory strategies in patients sharing similar baseline characteristics. Further details regarding these populations are given below and in Supplemental Methods.

Statistical analysis was performed using the R software (version 4.1.3) with packages *lme4*, *emmeans*, *lmerTest*, *pbkrtest*, *mice*, *MatchIt* and *survival* [17]*.* A *P* value < 0.05 was chosen for statistical significance. Data was expressed as median [1st quartile to 3rd quartile] for quantitative variables and counts (percentages) for categorical variables, respectively. Comparison between study groups of variables measured once was performed using the Wilcoxon-Mann–Whitney test for continuous variables, and the Fisher test for categorical variables.

Comparison between groups of longitudinally collected data (measured on Day 1, 3 and 7) was performed using mixed effects linear regression models, with the interaction of the study group and time as fixed effect (Group × Time). To explore the impact of each respiratory variable on E_L,PBW_ and MP_RS_, we performed a double stratification analysis in which one respiratory parameter was divided in quartiles of distinct mean values (e.g. ∆P) in subsamples of observations with matched mean levels of another variable of interest (e.g. P_PLAT_) [18].

Time to successful VV-ECMO weaning was evaluated using a univariate Cox model, and subsequently using univariate and multivariate Fine and Gray models accounting for the competing risk of death. Propensity score matching was also performed to compare clinical outcomes in a subset of patients sharing similar baseline characteristics (listed in Supplemental Methods). The full details of statistical methods are given in Supplemental Methods.

## Results

Population characteristics

**Fig. 1:**
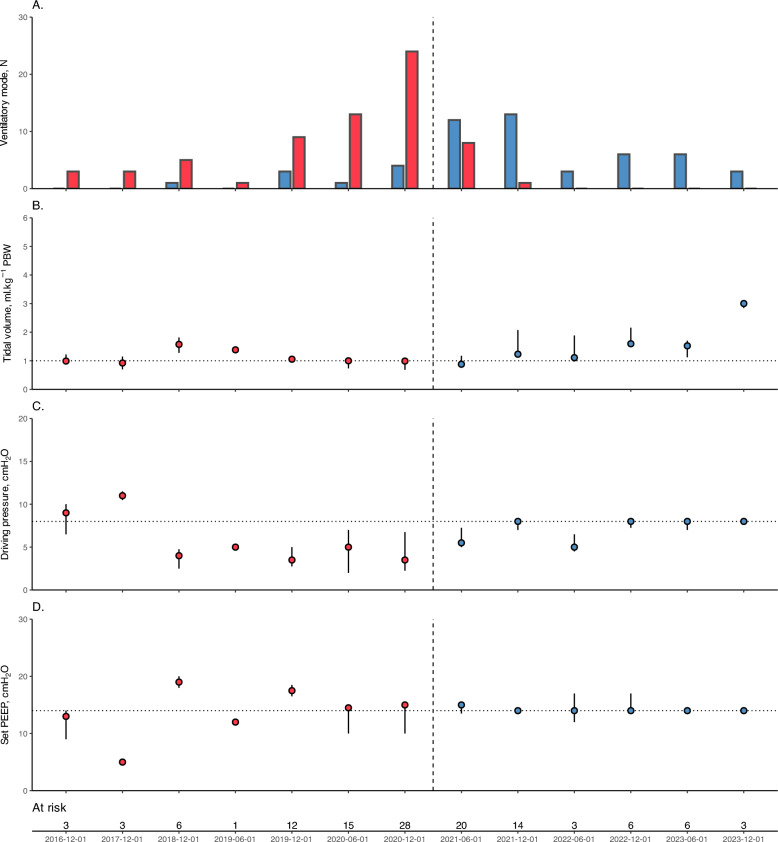
Ventilatory settings and respiratory mechanics over the study period. The figure shows the number of patients receiving each ventilatory mode (**A**), and the median (with interquartile range) of the measured tidal volume (**B**), measured driving pressure (**C**) and set PEEP (**D**), on day 1 after ECMO cannulation the study (from December 2016 to January 2024, divided into semesters). Red bars and dots representing patients ventilated receiving ACV ventilatory mode in panel A and the VT1 strategy in panels B to D; blue bars correspond to pressure-controlled modes in panel A, and ∆P8 strategy in panels B to D. The vertical dotted line represents the change in ventilatory strategy in June 2021. Below the panels are the number of patients at each time point. *ACV* assist-control volume, *∆P8* ultraprotective strategy in pressure mode with a driving pressure of 8 cmH_2_O, *PBW* predicted body weight, *PEEP* positive end-expiratory pressure, *VT1* quasi-apneic ventilatory strategy with a tidal volume of 1 ml.kg^−1^ predicted body weight

Between December 1st, 2016 and January 31st, 2024, 144 patients were screened and 121 were enrolled in the study, with 69 enrolled during the before period (VT1 strategy), and 52 in the after period (ΔP8 strategy) (Supplemental Figure 1). No patients were lost to follow-up. Figure 1 shows the ventilatory modes and settings, and respiratory mechanics used during both study periods. The main ARDS risk factor was viral pneumonia in 64% of cases (mainly COVID-19), and the principal indication for VV-ECMO was refractory hypoxemia (PaO_2_/FiO_2_ ratio before ECMO of 64 [55–74] mmHg, Table 1).

**Table 1:** Population characteristics at baseline

	All patients	VT1	∆P8	P value
Variables	N = 121	N = 69	N = 52	
Age, years	51 [41–61]	54 [47–62]	45 [35–60]	< 0.01
Sex, male, N (%)	74 (61%)	41 (59%)	33 (63%)	0.71
Body weight at ICU admission, kg	87 [74–98]	86 [74–96]	88 [74–105]	0.44
Body weight at time of inclusion, kg	85 [72–100]	86 [70–94]	85 [72–104]	0.58
Fluid balance at time of inclusion, kg	0 [-3–1]	0 [-1–2]	0 [-6–0]	0.03
Body mass index, kg.m^−2^	30 [26–34]	30 [26–34]	31 [26–36]	0.38
Comorbidities				
Diabetes, N (%)	25 (21%)	9 (13%)	16 (31%)	0.02
Chronic heart failure, N (%)	1 (1%)	1 (1%)	0 (0%)	> 0.9
Chronic kidney disease, N (%)	1 (1%)	0 (0%)	1 (2%)	0.43
Cancer, N (%)	1 (1%)	1 (1%)	0 (0%)	> 0.9
Hematologic malignancy, N (%)	8 (7%)	5 (7%)	3 (6%)	> 0.9
Medical admission category, N (%)	114 (94%)	62 (90%)	52 (100%)	0.02
SAPS-2 score at ICU admission	52 [36–61]	56 [38–62]	46 [34–56]	0.02
Total SOFA score at time of inclusion	9 [7–13]	8 [8–13]	9 [5–12]	0.76
ARDS risk factors				0.07
Viral pneumonia, N (%)	78 (64%)	50 (72%)	28 (54%)	
Bacterial pneumonia, N (%)	33 (27%)	14 (20%)	19 (37%)	
Aspiration pneumonia, N (%)	3 (2%)	1 (1%)	2 (4%)	
Other, N (%)	5 (3%)	3 (4%)	2 (2%)	
None identified, N (%)	1 (1%)	0 (0%)	1 (2%)	
COVID-19 viral pneumonia, N (%)	73 (60%)	45 (65%)	28 (54%)	0.26
VV-ECMO principal indication				< 0.01
Refractory hypercapnia, N (%)	18 (15%)	16 (23%)	2 (4%)	
Refractory hypoxemia, N (%)	102 (84%)	52 (75%)	50 (96%)	
Delay between ICU admission and intubation, days	1 [0–2]	1 [0–2]	1 [0–2]	0.83
Delay between intubation and VV-ECMO, days	3 [1–7]	4 [1–7]	3 [1–7]	0.37
Delay between ICU admission and VV-ECMO, days	5 [2–10]	6 [4–10]	4 [2–9]	0.48
ARDS adjunctive therapies at time of inclusion, before VV-ECMO				
Neuromuscular blockade, N (%)	120 (99%)	68 (99%)	52 (100%)	> 0.9
Inhaled nitric oxide, N (%)	76 (63%)	42 (61%)	34 (65%)	> 0.9
Prone positioning, N (%)	120 (99%)	68 (99%)	52 (100%)	> 0.9
Renal replacement therapy, N (%)	19 (16%)	9 (13%)	10 (19%)	0.45
Norepinephrine administration, N (%)	63 (52%)	36 (52%)	27 (52%)	0.85
Norepinephrine dose (tartrate), µg.min^−1^.kg^−1^	0.03 [0–0.49]	0.04 [0–0.48]	0.01 [0–0.44]	0.77
Arterial lactate concentration, mmol.L^−1^	1.9 [1.5–3]	1.9 [1.4–3]	2 [1.6–2.8]	0.53

Data is median [interquartile range] or count (percentage)

*ARDS* acute respiratory distress syndrome, *∆P8* ultraprotective strategy in pressure mode with a driving pressure of 8 cmH_2_O, *ICU* intensive care unit, *SAPS-2* simplified acute physiology score 2, *SOFA* sepsis-related organ failure assessment, *VT1* quasi-apneic ventilatory strategy with a tidal volume of 1 ml.kg^−1^predicted body weight, *VV-ECMO* veno-venous extracorporeal membrane oxygenation

### Impact of ventilatory strategies on respiratory mechanics and ECMO settings

**Fig. 2:**
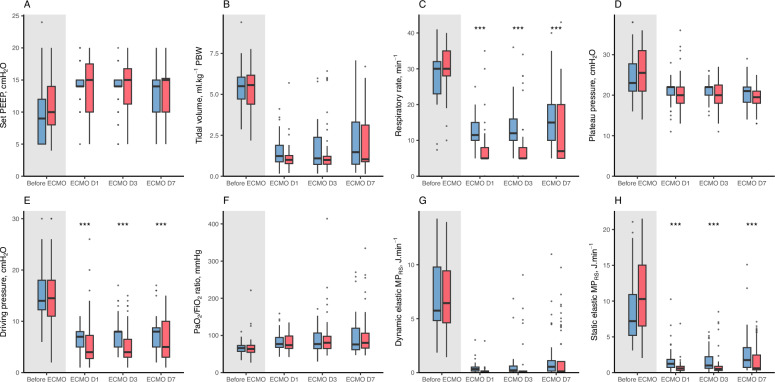
Respiratory mechanics over the first 7 days after VV-ECMO canulation in all patients. The figure shows the course over time of set PEEP (**A**), tidal volume (**B**), respiratory rate (**C**), plateau pressure (**D**), driving pressure (**E**), PaO_2_/FiO_2_ ratio (**F**), dynamic elastic mechanical power (**G**), static elastic mechanical power (**H**) in the patients receiving the VT1 strategy (in red) or the ΔP8 strategy (in blue). Day 1 corresponded to the day of ECMO cannulation. Data is represented by mean of boxplots (with median, first and third quartile) and outliers (black dots). The number of patients evaluated at each of the 4 time points was 121, 117, 105 and 83, respectively. For each studied variable, the difference between groups was evaluated using mixed effect regression models applied to measurements obtained on day-1 (D1), day-3 (D3) and day-7 (D7), with the interaction of Time × Group as the independent variable, and the patient identification code as the random intercept. In case of a significant interaction, a post-hoc pairwise comparison was performed between study groups, adjusted using the Sidak method. In case of a non-significant interaction term but with a significant effect of Group, the results of pairwise comparisons were given, using the Sidak method. There was no significant difference between study groups in all displayed variables at baseline. The *** in panels C, E and H indicate a significant difference at this time point between study groups (P < 0.05, non-significant interaction term). For all presented variables, there was a statistically significant difference between Pre-ECMO and Day 1 values (*P* < 0.05). *∆P8* ultraprotective strategy in pressure mode with a driving pressure of 8 cmH_2_O, *MP*_*RS*_ respiratory system mechanical power, *PaO*_*2*_*/FiO*_*2*_ ratio of arterial oxygen partial pressure to the fraction of inspired oxygen, *PBW* predicted body weight, *PEEP* positive end-expiratory pressure, *VV-ECMO* veno-venous extracorporeal membrane oxygenation, *VT1* quasi-apneic ventilatory strategy with a tidal volume of 1 ml.kg^−1^ predicted body weight

The application of both ventilatory strategies with VV-ECMO led a significant decrease in ∆P, P_PLAT_, V_T_ and RR, and higher applied PEEP, compared to the pre-ECMO period (Fig. 2). Compared to its pre-ECMO value, E_L,PBW_ significantly increased on ECMO day 1 (2.7 [2.2–3.8] vs. 4.3 [2.8–7.1] cmH_2_O.ml^−1^.kg^−1^ PBW, *P* < 0.01), with no difference between strategies.

During follow-up, the ΔP8 strategy was associated with a significantly higher RR, ∆P, static elastic MP_RS_ (Fig. 2), and lower PaCO_2_ and ECMO pump flow (Supplemental Table 1), as well as with a more frequent use of norepinephrine and less frequent prone positioning (Supplemental Fig. 2), compared to the VT1 strategy. Ventilatory modes are shown in Supplemental Fig. 3. Results were similar when assessing respiratory mechanics in patients receiving per-protocol ventilatory settings (Supplemental Figs. 4 and 5, Supplemental Table 2).

### Association of ventilatory settings with respiratory mechanics

**Fig. 3:**
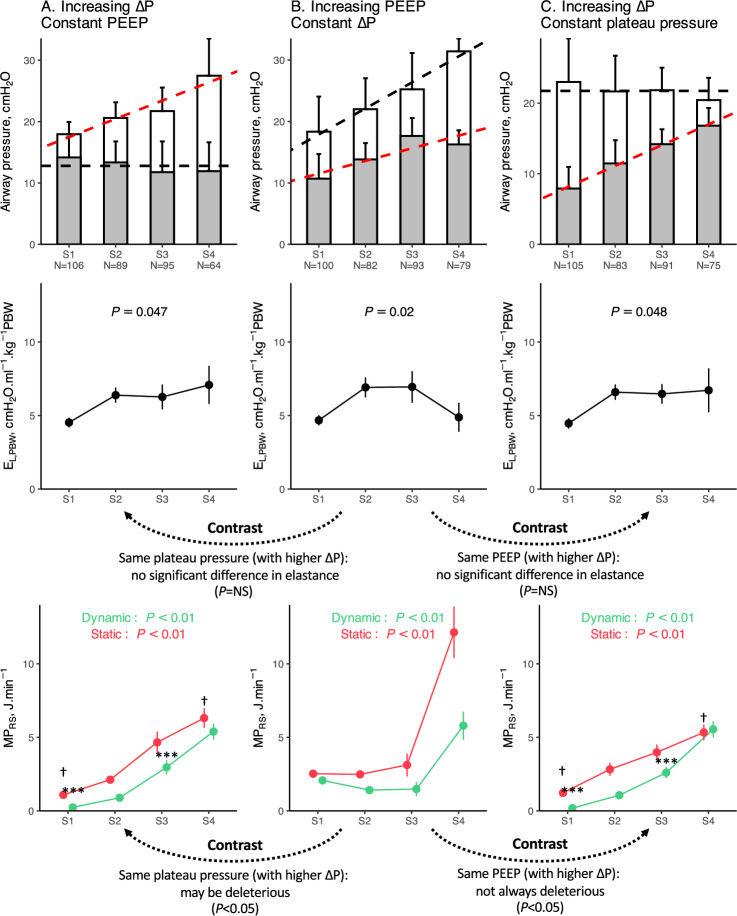
Effects on normalized elastance and mechanical power of increasing driving pressure and PEEP. Using double stratification (obtaining subgroups of observations with matched mean levels for one variable but different mean levels of another ranking variable), the figure shows the association of increasing driving pressure (columns A and B) and PEEP (columns B and C), in observations with similar levels of set PEEP (**A**), driving pressure (**B**) or plateau pressure (**C**). The first row depicts the effect size of airway pressures (mean value ± standard deviation) in each stratum (or subsample), and the number of observations in each stratum. The red dotted line corresponds to the slope of increasing driving pressure or PEEP, respectively. The black dotted lines correspond to the mean value of the other ranking variable (PEEP and plateau pressure). The second row shows the mean normalized elastance (± standard error) for each stratum. The third row shows the means values (± standard error) of static elastic (red points and lines) and dynamic elastic mechanical power (green points and lines) in each stratum. *P* values examine the association of normalized elastance on the one side, and mechanical power on the other, with strata (as a categorical variable, S1 being the reference level), using linear regression. Note that the subsample S4 in panel B demonstrated increased plateau pressure without an increase in PEEP, compared to the S3 strata (probably corresponding to pre-ECMO observations). Also, note that normalized elastance is mathematically related to driving pressure, and that PEEP and driving pressure are mathematically linked to mechanical power. The dotted arrows indicate the contrasts between subsamples A and B, and B and C, within each stratum, using pairwise comparison adjusted for multiple comparison. ***: significant difference in dynamic elastic mechanical power in this stratum in pairwise comparison between A and B, or B and C. †: significant difference in static elastic mechanical power in this stratum in pairwise comparison between A and B, or B and C. *∆P* driving pressure, *E*_*L,PBW*_ normalized elastance of the respiratory system, *MP*_*RS*_ respiratory system mechanical power, *PBW* predicted body weight, *PEEP* positive end-expiratory pressure

Higher pre-ECMO E_L,PBW_ was not significantly associated with higher ∆P or P_PLAT_ in the VT1 strategy, or lower V_T_ in the ∆P8 strategy (Supplemental Fig. 6). In the double stratification analysis, increasing ∆P at constant PEEP, or increasing PEEP at constant plateau, were significantly associated with higher E_L,PBW_ and higher static and dynamic elastic MP_RS_, while keeping ∆P constant only increased MP_RS_ in the highest stratum (Fig. 3).

Increasing V_T_, compared to increasing ∆P, demonstrated distinct patterns in their relationship with E_L,PBW_ (when P_PLAT_ was kept constant) but had similar effects on MP_RS_ values (Supplemental Fig. 7). Finally, increasing RR was not associated with a significant modification of E_L,PBW_ when studied in averaged samples of same V_T_ or ∆P, but was associated with higher values of MP_RS_.

### Effects of ventilatory strategies on quantitative CT findings

Quantitative CT scan results were available in 32 patients (15 with the VT1 strategy, 17 with the ΔP8 strategy). At time of CT study, patients of the ΔP8 strategy had significantly higher P_PLAT_ and RR, compared to the VT1 strategy (Supplemental Table 3). Quantitative CT showed no significant difference between groups in terms of non-inflated lung mass and aerated lung volume at PEEP 5 cmH_2_O, or in lung recruitment potential between PEEP 5 and PEEP 15 cmH_2_O.

### Impact of ventilatory strategies on time to successful ECMO weaning

**Fig. 4:**
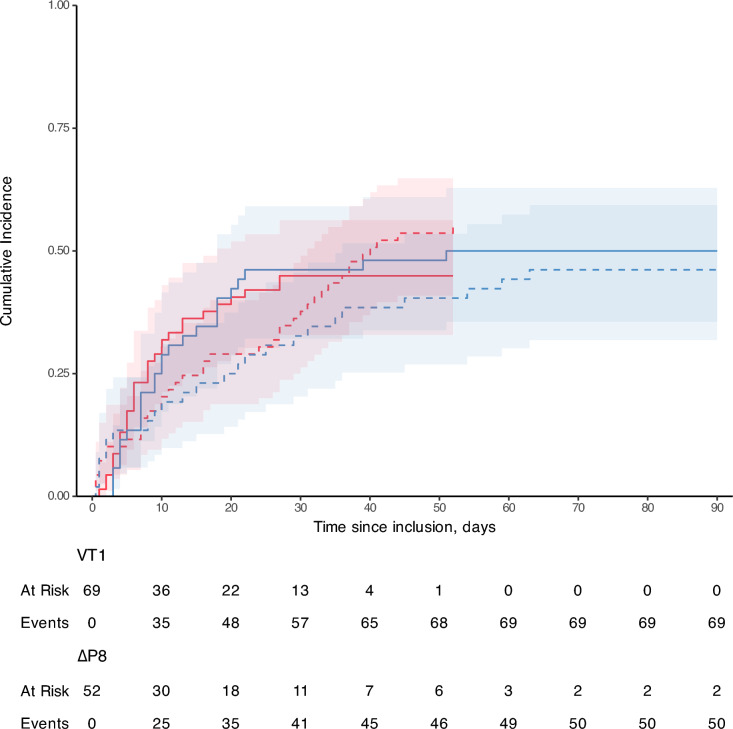
Time to successful ECMO weaning at day 90. The figure shows the time to successful VV-ECMO weaning in the whole cohort (broad lines), in patients of the before period (VT1 strategy, in red), and the after period (ΔP8 strategy, in blue). The represented cumulative incidence accounts for the competitive risk of death in the cohort (dashed lines). Patients alive and with VV-ECMO at day 90 were censored at this time. The red and blue shades depict the 95% interval of the cumulative incidences at a given time. The number of patients at-risk and with events are given below the figure. The red lines stop after day 50 because all patients in this group had met one or the other competitive outcomes (ECMO weaning or death). *∆P8* ultraprotective strategy in pressure mode with a driving pressure of 8 cmH_2_O, *VV-ECMO* veno-venous extracorporeal membrane oxygenation, *VT1* quasi-apneic ventilatory strategy with a tidal volume of 1 ml.kg^−1^ predicted body weight

Successful VV-ECMO weaning occurred in 57 patients (47%), with a time to event of 7 [4–13] days in 90-day survivors and no significant difference between study groups in clinical outcomes (Table 2). The hazard ratio of time to successful ECMO weaning with the ∆P8 strategy was 1.00 (95% confidence interval: [0.60–1.68], *P* > 0.9, univariate Cox analysis), compared to the VT1 strategy. The cumulative incidence of successful VV-ECMO weaning was not significantly different between study groups in univariate (Fig. 4) and multivariate competitive risk analysis (Supplemental Table 4). Results were unchanged in the propensity-matched subpopulation (N = 62, Supplemental Tables 5 and 6, and Supplemental Fig. 8).

**Table 2:** Whole population outcomes at day-90

	All patients	VT1	∆P8	P value
Variables	N = 121	N = 69	N = 52	
Alive at day-90, N (%)	43 (36%)	21 (30%)	22 (42%)	0.19
ECMO-free days at day-90, days	0 [0–77]	0 [0–77]	0 [0–79]	0.49
ECMO and vital status at day-90				0.32
Alive with ECMO, N (%)	2 (2%)	0 (0%)	2 (4%)	
Alive and ECMO-free, N (%)	41 (34%)	21 (30%)	20 (38%)	
Death while under ECMO, N (%)	62 (51%)	38 (55%)	24 (46%)	
Death after ECMO successful weaning, N (%)	16 (13%)	10 (14%)	6 (12%)	
Time to successful ECMO weaning in day-90 survivors, days	7 [4–13]	6 [4–11]	9 [6–18]	0.14
Alive and mechanical ventilation-free at day-90, N (%)	35 (29%)	16 (23%)	19 (37%)	0.50
Ventilator-free days at day-90, days	0 [0–41]	0 [0–0]	0 [0–67]	0.04
ICU length of stay in day-90 survivors, days	27 [21–58]	31 [21–47]	26 [20–64]	0.81
Hospital length of stay in day-90 survivors, days	58 [43–97]	66 [42–108]	52 [44–96]	0.64

Data is median [interquartile range] or count (percentage)

*∆P8* ultraprotective strategy in pressure mode with a driving pressure of 8 cmH_2_O, *ICU* intensive care unit, *ECMO* extracorporeal membrane oxygenation, *VT1* quasi-apneic ventilatory strategy with a tidal volume of 1 ml.kg^−1^ predicted body weight

## Discussion

In this single center, retrospective, before-and-after study, we evaluated the physiological and clinical effects of two protocolized ventilatory strategies applied to patients with ARDS and VV-ECMO. We observed that 1/ the protocolized ∆P8 strategy led to an increase in ∆P and an increase in MP_RS_ subcomponents (mainly driven by higher RR) and improved decarboxylation; 2/ both strategies were associated with a massive loss in aerated lung volumes and similar amount of lung recruitment potential in quantitative CT analysis, and 3/ the two strategies led to a similar delay to successful ECMO weaning;

It is a well admitted concept that ultra-protective ventilation, by limiting stress and strain, is a reasonable option when ventilating ARDS patients with VV-ECMO [6, 19]. Various ultraprotective strategies with or without extracorporeal support have been previously tested in clinical trials, yet with limited clinical benefits [5, 20, 21]. In the present study, both strategies drastically reduced ∆P, V_T_ and RR, well below those reported in recently published studies [22, 23]. As a consequence, MP_RS_ elastic subcomponents were considerably reduced compared to the pre-ECMO period, with significantly higher elastic MP_RS_ with the ∆P8 strategy being driven by higher RR and PEEP settings in this group.

On the other hand, we observed that keeping ∆P constant was associated with better control of MP_RS_ over varying ranges of P_PLAT_ and PEEP [18]. Indeed, the fixed ∆P setting in pressure mode led to a greater variability in applied V_T_ (reaching 4 ml.kg^−1^ PBW in some patients), while the fixed V_T_ setting in volume mode implied variability in ∆P values (with some values > 14 cmH_2_O), in relation with the underlying respiratory system compliance. Yet, it is physiologically sound to estimate that the ∆P8 strategy, by applying a V_T_ adjusted to respiratory system compliance in these severely ill cases, may prove more secure in limiting both barotrauma and volutrauma, as demonstrated in the double stratification analysis. However, with both strategies, these two determinants of cyclic stress and dynamic elastic MP_RS_ did not significantly differ, even when stratifying their assessment on the pre-ECMO E_L,PBW_.

Choosing a fixed PEEP of 14 cmH_2_O is debatable (although it was within the range of PEEP setting applied in the recent PRONECMO trial), when only half of patients with VV-ECMO demonstrated significant recruitment potential in the CT sub-analysis (i.e. > 5% of the total lung mass between PEEP 5 and 15 cmH_2_O) [24]. This may have exposed some patients to alveolar overdistension and to PEEP-induced hemodynamic impairment. On the other hand, the VT1 strategy mandated that P_PLAT_ be maintained between 20 and 25 cmH_2_O, which led to greater variability in PEEP setting, and potentially lead to the application of lower PEEP in patients with the lowest respiratory system compliance [25]. However, we did not observe significant differences in respiratory mechanics when patients were stratified based on their pre-ECMO E_L,PBW_ [26].

RR is a major equational determinant of MP_RS_ computation [27], and is associated with worse outcome [28]. Our data did not demonstrate an association between low RR and deteriorated elastance, evocative that that low-RR ventilation may not participate to alveolar derecruitment during ultra-protective ventilation (contrary to low pressure – low volume ventilation), while allowing a significant decrease in MP_RS_.

The quantitative CT substudy offers an insight on the consequences of these strategies on the lung parenchyma. First, and with both strategies, the non-aerated lung mass (a proxy of the lung’s loss of aeration) was high, and the aerated lung volume at PEEP 5 cmH_2_O (the *baby lung*) was nearly a quarter of that observed in severe ARDS without VV-ECMO [29]. Apart from the disease’s natural course, we hypothesize that massive aeration loss could be the consequence of low V_T_, through decreased tidal recruitment and despite high PEEP. Indeed, observations with low VT (whichever the strategy) were associated with the highest E_L,PBW_, although we cannot conclude whether this was the consequence of disease’s severity or of the ventilatory setting.

Both strategies led to a quasi-complete dependence of patients to the extracorporeal technique, which required applying high pump blood flows and increasing sweep gas flow rates over time. Additionally, the VT1 strategy led to higher PaCO_2_, consequential to a trend in lower minute ventilation, and was only partially compensated by the extracorporeal membrane.

This study has several strengths. Using standardized case report form, we report a large cohort, with longitudinal respiratory mechanics, collected in the context of protocolized ventilatory strategies. Moreover, we have combined these data to our quantitative CT scan database which gives further insight of the parenchymal effects of ultraprotective ventilation. Finally, we used adequate statistical methodology, including competitive risk regression models, propensity score matching, per-protocol analysis, and mixed effects linear regression to evaluate primary and secondary outcomes.

We also acknowledge several limitations. First, this is a single center, retrospective, observational, before-and-after study, which limits the generalizability of the results. Indeed, the study’s non-interventional design only allows drawing hypotheses on the physiological impact of strategies based on significant associations. Also, the 60% mortality rate observed in our cohort is above the numbers usually reported in past studies [5, 6], yet similar to the rates observed in COVID-19 severe ARDS [24, 30, 31] (in relation with the over-representation of this ARDS risk factor in our cohort) or in a recent meta-analysis [32]. Furthermore, the mortality rate of VV-ECMO ARDS may depend on the canulation strategy (liberal vs. conservative) and the pre-ECMO optimization strategies (fluid balance, systematic prone positioning). Second, designing groups based on the period of time may not reflect the exact ventilatory strategy received by a given patient, as some patients may have switched from a strategy to another, and given the absence of a washout period. However, the per-protocol analysis showed similar results in terms of respiratory physiology and clinical outcome. Yet, unreported confounders and potential imbalance between study groups may still have impacted our results. Third, more than two thirds of the cohort were patients with viral ARDS, whose respiratory mechanics were known to be less altered for the same level of hypoxemia compared to non-COVID-19 ARDS [33], and in which the quasi-apneic VT1 strategy might have been less physiologically relevant. Fourth, the results of the PRONECMO trial led to significant decrease in prone positioning use after the publication of the results, which may have impacted physiological and clinical outcomes [24]. Fifth, we did not collect ECMO-related complications, in relation with the difficulty to adequately collect these events retrospectively. Sixth, although the protocols mandated the evaluation of ECMO weaning potential daily after day 2, we were unable to report the rate of application of the weaning procedure. However, the fact that the median time to VV-ECMO weaning was 7 days in the cohort, lower than reported in previous studies [6], favors the hypothesis that it was adequately performed. Seventh, we did not report the rate of acute right ventricular failure or *acute cor pulmonale*, as this item was not systematically reported. Finally, although one may suspect that both strategies (especially the VT1 strategy) may have led to circuit humidification failure, we did not collect or report data regarding this specific adverse effect.

## Conclusions

In this single center, retrospective, before-and-after study, a protocolized pressure-controlled ventilation strategy with an 8 cmH_2_O ∆P, 14 cmH_2_O of PEEP and a RR of 10 min^−1^, led to the application of higher ∆P, RR and static elastic MP_RS_, lower VV-ECMO pump flow and improved decarboxylation, compared to a protocolized quasi-apneic ventilation strategy in ACV mode with a V_T_ of 1 ml.kg^−1^ PBW, a P_PLAT_ ≤ 25 cmH_2_O and a RR of 5 min^−1^, in patients with ARDS and VV-ECMO. Both strategies significantly reduced known determinants of ventilator-induced lung injuries, with none being associated with shorter time to successful VV-ECMO weaning.

## Supplementary Information

Additional file 1.

## Data Availability

Source datasets are not publicly available due to ethical reasons. Further enquiries can be directed to the corresponding author at laurent.bitker@chu-lyon.fr. The authors vouch for the accuracy and completeness of the data.

## Abbreviations

ACV: Assist-controlled volume
APRV: Airway pressure release ventilation
ARDS: Acute respiratory distress syndrome
BIPAP: Biphasic intermittent positive airway pressure
∆P8: Ultraprotective strategy in pressure mode with a driving pressure of 8 cmH_2_O
PBW: Predicted body weight
PEEP: Positive end-expiratory pressure
P_PLAT_: Plateau pressure
RR: Respiratory rate
VILI: Ventilator-induced lung injuries
V_T_: Tidal volume
VT1: Quasi-apneic ventilatory strategy with a tidal volume of 1 ml.kg^−^^1^ predicted body weight
VV-ECMO: Veno-venous extracorporeal membrane oxygenation
