# Supplementary material for: Physiological and clinical effects of two ultraprotective ventilation strategies in patients with veno-venous extracorporeal membrane oxygenation: the ECMOVENT study
Source: Ann Intensive Care. 2025 Aug 1;15:111. doi: 10.1186/s13613-025-01525-0 (PMC12316621; doi:10.1186/s13613-025-01525-0)
Supplement: Supplementary file 1 — Additional file 1. [file 13613_2025_1525_MOESM1_ESM.pdf]

Physiological and clinical effects of two ultraprotective ventilation strategies in patients with veno-venous extracorporeal membrane oxygenation: the ECMOVENT propensity-matched study

ONLINE SUPPLEMENT

Definition of per-protocol ventilatory strategies ... 2
Data collection and study time points ... 2
VV-ECMO eligibility, management and weaning ... 4
ARDS co-interventions and rescue strategies ... 5
Quantitative CT analysis ... 5
Statistics ... 6
SUPPLEMENTARY TABLES ... 10
Supplemental Table 1. Respiratory mechanics, cardiovascular support and ECMO settings over the first 7 days, in all patients ... 10
Supplemental Table 2. Respiratory mechanics and ECMO settings over the first 7 days, in patients with per-protocol ventilation application ... 13
Supplemental Table 3. Quantitative CT study ... 15
Supplemental Table 4. Univariate and multivariate analyses of variables associated with ECMO successful weaning ... 16
Supplemental Table 5. Matched population characteristics at baseline and clinical outcomes ... 18
Supplemental Table 6. Matched vs discarded population characteristics at baseline and outcomes at day 90 ... 20
SUPPLEMENTARY FIGURES ... 22
Supplemental Figure 1. Study flowchart ... 22
Supplemental Figure 2. Rate of use of neuromuscular blockade and prone positioning in all patients over the first 7 days of VV-ECMO ... 23
Supplemental Figure 3. Ventilatory mode used over time in all patients over the first 7 days of VV-ECMO 24
Supplemental Figure 4. Application of ventilatory strategies in all patients on day 1 of VV-ECMO ... 25
Supplemental Figure 5. Respiratory mechanics over the first 7 days after VV-ECMO canulation in patients receiving per-protocol ventilation ... 26
Supplemental Figure 6. Respiratory mechanics on the first day of ECMO canulation in all patients, as a function of the normalized elastance before ECMO canulation ... 28
Supplemental Figure 7. Effects on normalized elastance and mechanical power of increasing tidal volumes and respiratory rates ... 30
Supplemental Figure 8. Time to successful ECMO weaning in matched patients alive at day 90 ... 32
SUPPLEMENTAL REFERENCES ... 34

Supplementary methods

Definition of per-protocol ventilatory strategies

The items defining per-protocol ventilation are summarized in the table below:

Criteria defining per-protocol application of ventilatory strategies during veno-venous ECMO

	**VT1 strategy** (4 criteria)	**ΔP8 strategy** (5 criteria)
Ventilatory mode	ACV	BIPAP or APRV
Tidal volume, $ml.kg^{-1}$ PBW	< 1.5 (1)	-
Driving pressure, cmH_2O	-	7 to 9 (8)
Set PEEP, cmH_2O	-	≥ 13 (14)
Plateau pressure, cmH_2O	≤ 25 (20 to 25)	≤ 24 (24)
Respiratory rate, min^{-1}	≤ 6 (5)	≤ 11 (10)

On each study day, in patients still under ECMO, the respiratory setting or parameter was collected twice, corresponding to the observed values at the time of blood gas results showing the lowest and highest PaO_2/FiO_2 ratio, respectively. Fulfilment of per-protocol ventilation criteria were adjudicated using the lowest value of each parameter (tidal volume, set PEEP, plateau pressure and respiratory rate). A tolerance threshold of 1 cmH_2O for driving pressure and set PEEP was applied, as well as a 1 min^{-1} tolerance threshold for respiratory rates. Similarly, a tolerance of 0.5 $ml.kg^{-1}$ of tidal volume was deemed acceptable. The values recommended by the protocol are indicated in parenthesis. ΔP8: ultraprotective strategy in pressure mode with a driving pressure of 8 cmH_2O; ECMO: extracorporeal membrane oxygenation; ACV: assist-controlled volume ventilation; APRV: airway pressure release ventilation; BIPAP: biphasic intermittent positive airway pressure; PBW: predicted body weight; PEEP: positive end-expiratory pressure; VT1: quasi-apneic ventilatory strategy with a tidal volume of 1 $ml.kg^{-1}$ predicted body weight

We *a priori* defined per-protocol ventilation if any combination of 3 out 4 per-protocol criteria were present during the VT1 period, and 4 out of 5 during the ΔP8 period, on day 1 of ECMO run. Adhesion to ventilatory protocols is shown in **Supplemental Figure 4**.

Data collection and study time points

We retrospectively collected the following data in normalized case report forms: demographics, morphometrics, ARDS risk factors [1], comorbidities, acute physiology score (SAPS2) [2], ECMO date of canulation and indications, vital status at ICU discharge, date of ICU and hospital discharge, date of ECMO and invasive mechanical ventilation weaning.

The longitudinal data was collected on the following calendar days:

- Day 1 (ECMO canulation day): in the 2h preceding ECMO canulation
- Day 1 (ECMO canulation day): from the time of ECMO canulation to midnight of that calendar day
- Day 3 after canulation (from midnight to midnight)
- Day 7 after canulation (from midnight to midnight)

and corresponded to: ventilatory settings, respiratory mechanics, arterial blood gas, ARDS adjunctive treatments, norepinephrine dose (tartrate formulation), ECMO settings and SOFA scores [3]. Because there was multiple values of ventilatory settings, respiratory mechanics, arterial blood gas results and ECMO settings on a study day, we collected these corresponding to the blood gas showing the lowest PaO_2/FiO_2 ratio.

Norepinephrine dose and SOFA scores were collected at 8h±2h AM on the corresponding calendar day. ARDS adjunctive treatments included the presence, at any time during each calendar day, of nitric oxide, NMB, prone positioning and/or renal replacement therapy. Fluid balance was computed as the difference in weights measured on ICU admission day and on VV-ECMO canulation day.

On a given study day, collected respiratory mechanics and ventilatory settings corresponded to that observed (within a ±2h frame) closest to the blood gas results with the lowest PaO_2/FiO_2 ratio on the calendar day. Tidal volumes were reported in $ml.kg^{-1}$ PBW, using the ARDS network formula [4]. Total PEEP and plateau pressure were collected in ICU electronic charts by nurses trained to perform 3-sec expiratory and inspiratory pauses in patients without ventilator interactions. ΔP corresponded to the difference between plateau pressure and total PEEP. In case of a missing total PEEP measurement, ΔP was computed using the set PEEP. The respiratory system elastance corresponded to the driving pressure divided by the tidal volume (in ml) and was normalized to $cmH_2O.ml^{-1}.kg^{-1}$ PBW:

$$Normalized\ elastance_{cmH2O/ml/kg\ PBW} = \frac{Driving\ pressure_{cmH2O}}{Tidal\ volume_{ml/kg\ PBW}}$$

Normalized elastance was further classified in low (≤ 2 $cmH_2O.ml^{-1}.kg^{-1}$ PBW), intermediate (between 2 and 3 $cmH_2O.ml^{-1}.kg^{-1}$ PBW) or high (>3 $cmH_2O.ml^{-1}.kg^{-1}$ PBW) normalized elastance (13). Dynamic and static elastic mechanic powers were computed as follows:

$$MP_{RS,static} = 0.096 \times V_T \times PEEP_{tot} \times RR$$

$$MP_{RS,dynamic} = 0.096 \times V_T^2 \times EL_{RS} \times 0.5 \times RR$$

with $PEEP_{TOT}$ the total PEEP, RR the respiratory rate, EL_{RS} the respiratory system elastance [5,6]. In case of a missing total PEEP, the set PEEP was used.

VV-ECMO eligibility, management and weaning

Patients were deemed not eligible for VV-ECMO if they had one of the following conditions: disease with an expected survival < 5 years, cardiogenic shock (veno-arterial ECMO), decision to withdraw therapies, a sepsis-related organ failure assessment (SOFA) score > 18, age > 75 years, duration of mechanical ventilation (including the non-invasive ventilation period prior to intubation) > 10 days, end-stage chronic respiratory disease with no lung transplantation project, hematologic or solid organ malignancy with metastatic evolution under treatment, or cardiac arrest. Also, during the COVID-19 pandemic, French healthcare authorities emitted recommendations regarding VV-ECMO use in the context of high strain on available resources, and suggested contraindicating VV-ECMO in patients with a duration of mechanical ventilation > 7 days, or an age > 65 years. Finally, all VV-ECMO indications and non-indications were systematically discussed by two or more senior ICU physicians with VV-ECMO expertise.

Implantation of ECMO cannulas was performed percutaneously with femoro-jugular access. Rotaflow® (Getinge, Rastatt, Germany), Cardiohelp® (Maquet, Rastatt, Germany), or Xenios® (Fresenius Medical Care, Savigny, France) ECMO consoles were used, with one of the following membrane oxygenators: Alone® (Euroset, Medolla, Italy), PLS® (Maquet, Rastatt, Germany), HLS Set Advanced® (Maquet, Rastatt, Germany), or NOVALUNG XLung® (Fresenius Medical Care, Savigny, France). Anticoagulation under ECMO was performed with continuous unfractionated heparin targeting an anti-Xa activity between 0.2 and 0.3 $IU.ml^{-1}$.

A 2-hour protocolized ECMO weaning trial was performed daily. To do so, patients were switched to conventional lung protective ventilation, with progressive increase in V_T up to 6 $mL.kg^{-1}$ PBW while keeping plateau pressure < 25 cmH_2O, PEEP 10 cmH_2O and respiratory rate ≤ 35 min^{-1}. Furthermore, during the after period, the patient had to have an expired $V_T \geq$ 3 $ml.kg^{-1}$ PBW with a driving pressure of 8 cmH_2O to start the weaning trial. ECMO sweep gas

flow was set to 0 $L.min^{-1}$ during the weaning trial. ECMO weaning trial was deemed successful if PaO_2/FiO_2 was > 150 mmHg in the supine position, $PaCO_2$ < 50 mmHg, FiO_2 ≤ 60%, and plateau pressure < 25 cmH_2O after 2 hours. If these weaning criteria were not met, ultraprotective ventilation was resumed. The proper application of the daily weaning protocol was supervised by the unit's ECMo team to ensure adhesion.

ARDS co-interventions and rescue strategies

With both strategies, recruitment maneuvers were not recommended. Prone position sessions during VV-ECMO, of at least 16h, were initially recommended in all patients with VV-ECMO and PaO_2/FiO_2 ratio < 150 mmHg, until the results of the PRONECMO trial [7] were published, which led to a change in practice which recommended using prone positioning only in selected patients with dependent bilateral alveolar collapse and no contraindications to proning [8]. Sedation was performed with continuous IV midazolam infusion (targeting a score of -3 to -4 on the Richmond Agitation Sedation Scale [RASS]) [9] and analgesia was provided by continuous IV morphine infusion. If PaO_2/FiO_2 ratio was < 150 mmHg, NMB were added according to ACURASYS trial, for a maximal duration of 48 hours after ARDS identification [10], or in case of significant ventilatory asynchronies. Corticosteroids were considered in persistent ARDS [11]. Therapeutic hypothermia (34-36°C) were used only in case of refractory hypoxemia despite VV-ECMO.

Quantitative CT analysis

Some patients underwent a quantitative CT scan study comprising 4 low-dose CTs being performed at the following respiratory times: at end-expiration at the PEEP set by the clinician, at end-expiration at PEEP 5 cmH_2O, and at end-expiration at PEEP 15 cmH_2O. CT scans were then manually segmented by an expert [12], and radiomics (described below) were obtained using X-ray attenuation to quantify lung parenchymal density and aeration analyzed using Matlab routines. Tissue and gas volumes were computed as the product of their respective fractions by the voxel volume and the number of voxels in the segmented lung volume, respectively. The non-inflated lung mass was estimated using lung tissue volume of the non-inflated compartment (with a tissue density between -100 and +100 Hounsfield units),

assuming a tissue density of 1 g.mL^{-1}. Tidal hyperinflation corresponded to the hyperinflated volume (tissue density between -1000 and -900 Hounsfield units) difference between end-expiration and end-inspiration at the clinician's PEEP and was normalized to PBW. Lung recruitment corresponded to the difference in lung tissue mass of the non-aerated compartment between PEEP 15 cmH_2O and PEEP 5 cmH_2O.

Statistics

Missing baseline values were imputed on 10 datasets, using predictive mean matching of scaled continuous variables and logistic regression for categorical variables.

Quality of imputation of missing continuous variables

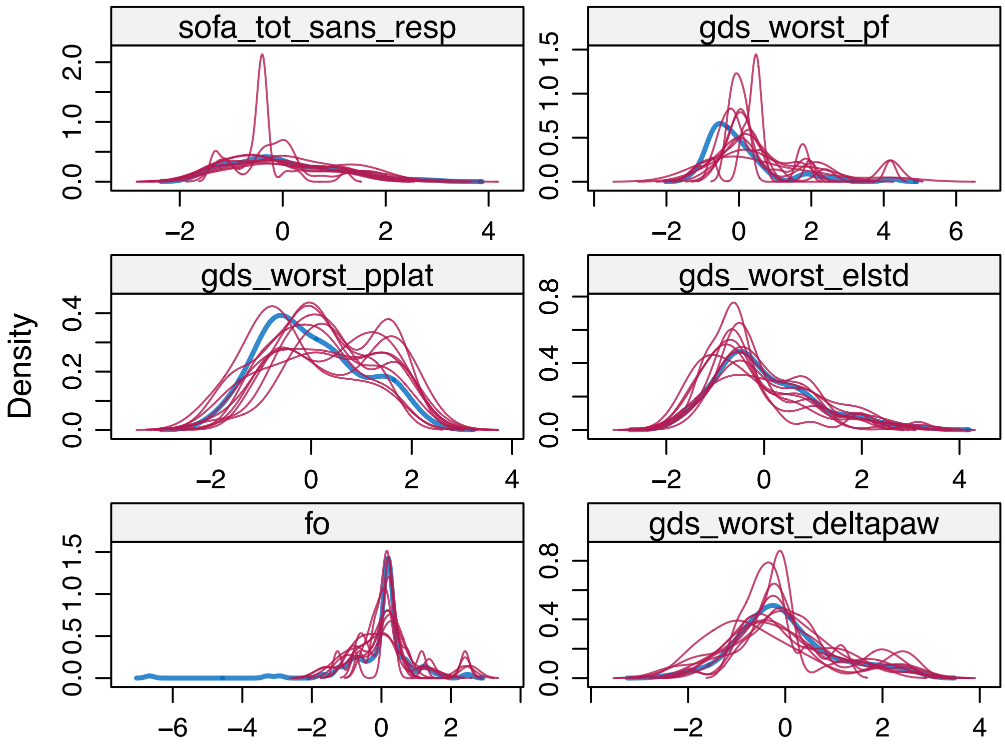

The figure shows the rate of missing values in the dataset (panel A) and the distribution of imputed variables in the 10 datasets (panel B, blue lines are observed distribution and red lines imputed ones) generated using predictive mean matching and logistic regression. In panel A, the numbers on the left-hand side are the number of variables with N missing variables, and on the right-hand side, the corresponding number of missing variables. In panel B, the 6 continuous variables with missing values at baseline were total SOFA score (1st subpanel), PaO_2/FiO_2 ratio (2nd subpanel), plateau pressure (3rd subpanel), normalized elastance (4th subpanel), fluid balance on ECMO cannulation day (5th subpanel), and driving pressure (6th subpanel). ECMO: extracorporeal membrane oxygenation; FiO_2: inspired fraction in O_2; PaO_2: arterial partial pressure in O_2

Comparison between groups of longitudinal data (measured on day 1, 3 and 7) was performed using mixed effects linear regression models, with the interaction of the study group and time as fixed effect (Group × Time), and the patient identification code as the random intercept (to account for the repetition of measurements in a given patient). In case of a non-significant interaction term but with a significant effect of Group, the results of pairwise comparisons were given, using the Sidak method. In case of a significant interaction term, a post-hoc pairwise analysis was performed to compare study groups at each time point, with adjustment for the repetition of tests using the Sidak method.

Evaluation of the impact of each ventilatory parameter of interest (driving pressure, tidal volume, PEEP, plateau pressure and respiratory rate) on normalized elastance (an indicator of the loss of lung aeration normalized to predicted body weight) and both components of elastic mechanical power (a summary metric associated with VILI generation) was performed using a double stratification method [13]. Using all available observations (from pre-ECMO to day 7), the observations were first stratified in quartiles of a respiratory parameter of interest (e.g. plateau pressure). Then, each subsample was re-stratified on quartiles of a second respiratory parameter of interest (e.g. driving pressure), leading to the production of 16 distinct subsamples. Finally, subsamples were remerged, and evaluated based on the second level of stratification. Six analyses were consequently produced:

- 4 sub-samples (or strata) with matched average PEEP, but distinct average ΔP (and plateau pressure)
- 4 sub-samples (or strata) with matched average driving pressure, but distinct average PEEP (and plateau pressure)
- 4 sub-samples (or strata) with matched average plateau pressure, but distinct average PEEP (and driving pressure)
- 4 sub-samples (or strata) with matched average plateau pressure, but distinct average VT
- 4 sub-samples (or strata) with matched average driving pressure, but distinct average respiratory rate

- 4 sub-samples (or strata) with matched average VT, but distinct average respiratory rate

The difference in normalized elastance and mechanical power between the 4 strata were then evaluated using linear regression (using strata as categorical variables and the first stratum as the reference level). Finally, pairwise comparisons between similar strata of different matched subsamples using adjustments for multiple comparisons (Tukey's method).

Time to successful ECMO weaning first evaluated by mean of a univariate Cox model (as recommended and despite the existence of a potential competitive risk), with the time to successful ECMO weaning as the outcome measure, and the study group as the explanatory variable. Then, a Fine and Gray competitive risk regression was performed with the study primary outcome as the outcome measure (i.e. successful weaning from VV-ECMO), and, accounting for the competitive risk of death, using the study group as the explanatory variable. Variables deemed physiologically relevant (age, gender, body mass index, viral pneumonia as the primary ARDS risk factor, SAPS-2 score at ICU admission [with removal of points related to age], non-respiratory SOFA score on ECMO canulation day, plateau pressure, PaO_2/FiO_2 ratio and normalized elastance prior to ECMO canulation, fluid balance on ECMO canulation day, delay between intubation and ECMO canulation, study group and elapsed time since date of enrolment of the first patient) were inserted into a multivariate model built on imputed datasets, and a backward stepwise selection procedure was performed to identify independent variables significantly associated with the outcome. Variables were scaled prior to regression. Multicollinearity was systematically ruled out, and interaction checked for in the final model. Schoenfeld's residuals were checked to test the proportional hazard assumption.

Other clinical outcomes were also compared in a subset of patients matched using a propensity score (1:1 ratio, nearest neighbor with no replacement, caliper of 0.15 of the standard deviation of the logit of the propensity score) determined using the covariate balancing propensity score method (CBPS) with the following balancing covariates: age, gender, delay between ICU admission and ECMO canulation, SAPS-2 score, fluid balance at inclusion, ECMO indication (refractory hypoxemia vs. refractory hypercapnia), ARDS risk factor

was viral pneumonia (vs. all other causes), admission category (medical vs. surgical) and pre-ECMO normalized elastance. In case of conjunct viral and bacterial infectious ARDS risk factors, bacterial was prioritized over viral. Covariates were scaled prior to propensity score determination. Quality of propensity matching was assessed using the mean standard differences and the variance ratios.

Quality of propensity score matching

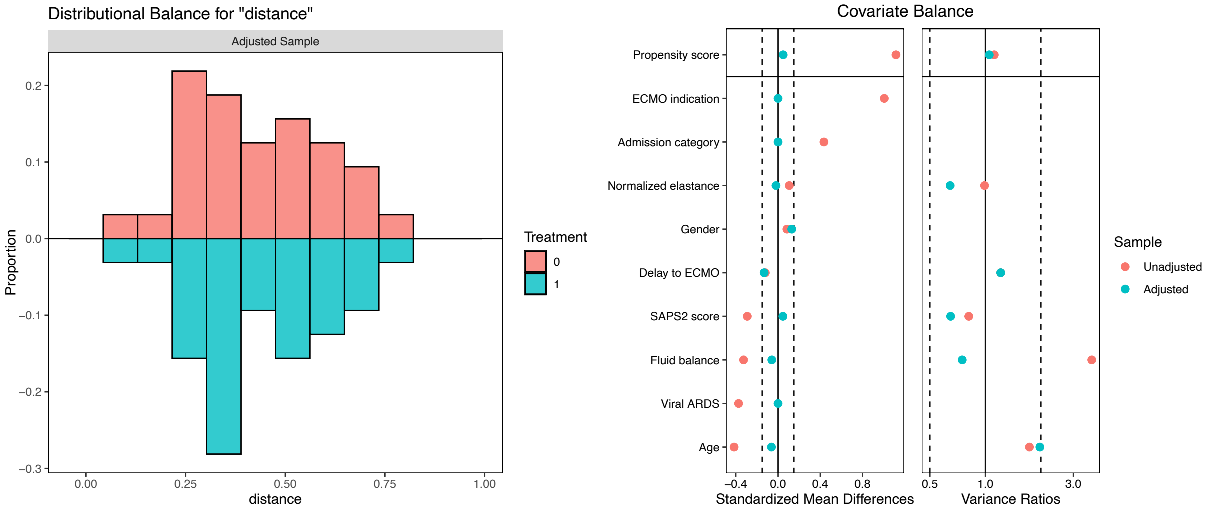

The first panel shows the distribution of propensity scores (i.e. distance) in the 2 study groups (VT1 in red, ΔP8 in blue). The propensity score was determined using covariate balancing propensity score method, with the following covariates: age, gender, delay between ICU admission and ECMO canulation, SAPS-2 score, fluid balance at inclusion, ECMO indication, ARDS risk factor (viral vs. any other risk factors) and admission category (medical vs. surgical). The second panel shows the standardized mean difference of balancing covariates before (in red) and after adjustment (in blue) on the left (with the dotted vertical line showing a caliper of ±0.15; and the variance ratio of continuous covariates before and after adjustment on the right (with a 0.5 to 2.0 range in broken lines). ARDS: acute respiratory distress syndrome; ΔP8: ultraprotective strategy in pressure mode with a driving pressure of 8 cmH_2O; ICU: intensive care unit; SAPS-2: simplified acute physiology score 2; SOFA: sepsis-related organ failure assessment; VT1: quasi-apneic ventilatory strategy with a tidal volume of 1 $ml.kg^{-1}$ predicted body weight; VV-ECMO: veno-venous extracorporeal membrane oxygenation

Supplementary tables

Supplemental Table 1. Respiratory mechanics, cardiovascular support and ECMO settings over the first 7 days, in all patients

Variable	Time	N at risk	All patients	VT1	ΔP8	P value		
						Group × Time	Group	Time
**Minute ventilation, L.min^{-1}**								
	Pre-ECMO	121	9.6 [7.7–11.7]	9.7 [7.7–11.8]	8.5 [7.6–11]	0.55	0.30	<0.01
	Day-1	117	0.5 [0.3–0.9]	0.4 [0.3–0.5]	0.9 [0.5–1.4]			
	Day-3	105	0.5 [0.3–1.2]	0.4 [0.2–0.6]	0.8 [0.5–1.9]			
	Day-7	83	0.8 [0.3–5.5]	0.4 [0.3–6.4]	1.8 [0.6–2.9]			
**$PaCO_2$, mmHg**								
	Pre-ECMO	121	58 [49–72]	60 [49–76]	57 [50–67]	0.92	<0.01	0.96
	Day-1	117	45 [41–52]	47 [44–57]	42 [38–46]			
	Day-3	105	46 [41–52]	50 [42–55]	45 [41–47]			
	Day-7	83	46 [43–50]	46 [42–53]	46 [43–49]			
**Respiratory system normalized elastance, $cmH_2O.ml^{-1}.kg^{-1}$ PBW**								
	Pre-ECMO	121	2.7 [2.2–3.8]	2.7 [2.3–3.7]	2.8 [2.2–4]	0.11	0.88	0.19
	Day-1	117	4.3 [2.8–7.1]	4.2 [2.6–7.6]	4.3 [3.5–6.1]			
	Day-3	105	5.2 [2.7–7.8]	4 [2.5–6.2]	6.5 [4.1–8.5]			
	Day-7	83	3.3 [2.2–6.2]	3.1 [2.1–5.7]	3.5 [2.5–6.5]			
**Mean airway pressure, cmH_2O**								
	Pre-ECMO	121	12 [10–15]	13 [11–15]	11 [9–15]	0.42	0.76	0.17
	Day-1	117	17 [15–17]	16 [14–18]	17 [16–17]			
	Day-3	105	16 [15–17]	16 [16–18]	17 [15–17]			
	Day-7	83	17 [12–17]	16 [12–18]	17 [13–17]			

**Norepinephrine, N (%)**						0.19	<0.01	<0.01
	Pre-ECMO	121	67 (50%)	40 (49%)	27 (52%)			
	Day-1	117	77 (59%)	41 (50%)	36 (69%)			
	Day-3	105	57 (48%)	33 (40%)	24 (46%)			
	Day-7	83	48 (41%)	23 (28%)	25 (48%)			
**Norepinephrine, µg.kg^{-1}.min^{-}1 (tartrate)**						0.64	0.095	<0.01
	Pre-ECMO	121	0.02 [0.00–0.48]	0.02 [0.00–0.47]	0.01 [0.00–0.45]			
	Day-1	117	0.10 [0.00–0.72]	0.04 [0.00–0.55]	0.13 [0.02–0.72]			
	Day-3	105	0.00 [0.00–0.12]	0.00 [0.00–0.09]	0.03 [0.00–0.17]			
	Day-7	83	0.00 [0.00–0.13]	0.00 [0.00–0.07]	0.03 [0.00–0.46]			
**Arterial lactate, mmol.L^{-1}**						>0.9	0.20	0.02
	Pre-ECMO	121	1.9 [1.5–3.0]	1.9 [1.4–3.0]	2.0 [1.6–2.8]			
	Day-1	117	2.2 [1.7–3.8]	2.2 [1.8–4.0]	2.0 [1.6–3.4]			
	Day-3	105	1.8 [1.4–2.5]	1.9 [1.4–2.3]	1.8 [1.5–2.6]			
	Day-7	83	2.1 [1.5–2.5]	2.2 [1.6–2.5]	2.0 [1.5–2.5]			
**ECMO pump flow rate, L.min^{-1}**								
	At canulation	121	4.2 [3.6–5.0]	4.3 [3.9–5.0]	4.0 [3.4–4.9]	0.27	0.02	0.07
	Day-1	117	4.0 [3.5–4.8]	4.3 [3.9–4.9]	3.6 [3.2–4.4]			
	Day-3	105	3.9 [3.3–4.5]	4.1 [3.7–4.7]	3.5 [3.1–4.1]			
	Day-7	83	3.9 [3.2–4.9]	4.0 [3.6–4.8]	3.5 [3.1–5.0]			
**ECMO sweep gas flow rate, L.min^{-1}**								
	At canulation	121	4 [4–5]	5 [4–5]	4 [3–5]	0.52	0.27	<0.01
	Day-1	117	5 [4–7]	5 [4–7]	5 [4–7]			
	Day-3	105	7 [5–8]	7 [6–9]	7 [5–8]			
	Day-7	83	8 [6–10]	8 [6–10]	7 [6–10]			

**ECMO sweep gas flow to pump flow ratio**								
	At canulation	121	1.0 [0.9–1.2]	1.1 [0.8–1.4]	1.0 [0.9–1.1]	0.16	0.96	<0.01
	Day-1	117	1.3 [1.1–1.8]	1.3 [1.0–1.7]	1.4 [1.1–1.9]			
	Day-3	105	1.7 [1.3–2.1]	1.8 [1.3–2.2]	1.7 [1.4–2.1]			
	Day-7	83	1.9 [1.5–2.3]	2.0 [1.5–2.5]	1.9 [1.5–2.3]			
**ECMO membrane oxygen fraction, %**								
	At canulation	121	100 [80–100]	90 [70–100]	100 [90–100]	0.40	0.82	0.07
	Day-1	117	90 [70–100]	85 [70–100]	90 [80–100]			
	Day-3	105	85 [70–100]	80 [70–90]	90 [68–100]			
	Day-7	83	80 [70–100]	80 [75–100]	90 [70–100]			

Data is median [interquartile range] or count (percentage).

Data corresponds to all included patients, alive at the given time point. *P* values evaluate the difference between study groups of the variable of interest measured on day-1, day-3 and day-7, using a mixed effects linear regression model, with the interaction term of Group × Time as a fixed effect, and the patient identification code as the random effect to account for the repetition of measurements. In case of a non-significant interaction, the independent association of Group and Time was evaluated. There was no significant difference between study groups during the pre-ECMO period.

ΔP8: ultraprotective strategy in pressure mode with a driving pressure of 8 cmH_2O; ECMO: extracorporeal membrane oxygenation; $PaCO_2$: arterial partial pressure in CO_2; PBW: predicted body weight; VT1: quasi-apneic ventilatory strategy with a tidal volume of 1 $ml.kg^{-1}$ predicted body weight

Supplemental Table 2. Respiratory mechanics and ECMO settings over the first 7 days, in patients with per-protocol ventilation application

						P value		
**Variable**	**Time**	**N at risk**	**All patients**	**VT1**	**ΔP8**	Group × Time	Group	Time
**Minute ventilation, L.min^{-1}**								
	Pre-ECMO	90	9.7 [7.9–12]	9.7 [7.7–11.9]	9.9 [8–11.9]	0.63	0.55	<0.01
	Day-1	90	0.5 [0.3–0.8]	0.4 [0.2–0.5]	0.9 [0.4–1.7]			
	Day-3	80	0.5 [0.3–1.1]	0.4 [0.2–0.6]	0.8 [0.5–2.5]			
	Day-7	61	0.6 [0.3–6.4]	0.4 [0.3–7.3]	1.3 [0.6–2.5]			
**$PaCO_2$, mmHg**								
	Pre-ECMO	90	58 [49–72]	62 [49–78]	56 [50–65]	0.31	<0.01	0.38
	Day-1	90	45 [41–51]	47 [44–57]	41 [38–45]			
	Day-3	80	46 [41–52]	50 [43–54]	43 [40–46]			
	Day-7	61	47 [43–51]	47 [42–52]	47 [44–49]			
**Respiratory system normalized elastance, $cmH_2O.ml^{-1}.kg^{-1}$ PBW**								
	Pre-ECMO	90	2.8 [2.3–3.9]	2.7 [2.2–3.7]	2.8 [2.3–4]	0.21	0.72	0.41
	Day-1	90	4.3 [2.7–6.9]	4 [2.3–7.1]	4.7 [3.2–6.4]			
	Day-3	80	4.4 [2.6–8.1]	3.4 [2.4–5.8]	7.1 [5.3–8.6]			
	Day-7	61	3.4 [2.2–6.3]	3 [2.1–5.4]	4.9 [2.8–6.6]			
**Mean airway pressure, cmH_2O**								
	Pre-ECMO	90	12 [11–15]	13 [11–15]	11 [10–14]	0.36	0.36	0.14
	Day-1	90	17 [16–17]	16 [14–17]	17 [16–17]			
	Day-3	80	16 [15–17]	16 [15–18]	17 [15–17]			
	Day-7	61	16 [12–17]	16 [11–18]	17 [14–17]			
**ECMO pump flow rate, L.min^{-1}**								
	At canulation	90	4.2 [3.6–4.9]	4.3 [3.8–5]	3.8 [3.3–4.4]	0.04		

Day-1	90	4 [3.5–4.7]	4.3 [3.9–5]	3.5 [3.1–3.8]*			
Day-3	80	3.9 [3.3–4.4]	4.1 [3.7–4.7]	3.4 [3.1–3.9]*			
Day-7	61	3.9 [3.2–4.7]	4 [3.7–4.8]	3.5 [3.2–4.4]			
**ECMO sweep gas flow rate, $L.min^{-1}$**							
At canulation	90	4 [4–5]	5 [4–5]	4 [3–5]	0.31	0.37	<0.01
Day-1	90	6 [4–7]	5 [4–7]	6 [4–7]			
Day-3	80	7 [6–8]	7 [6–9]	7 [5–7]			
Day-7	61	8 [6–10]	8 [6–10]	7 [6–9]			
**ECMO sweep gas flow to pump flow ratio**							
At canulation	90	1 [0.8–1.2]	1.1 [0.8–1.3]	1 [0.9–1.2]	0.12	0.39	<0.01
Day-1	90	1.4 [1.1–1.9]	1.3 [1–1.7]	1.5 [1.2–2.2]			
Day-3	80	1.8 [1.4–2.3]	1.8 [1.3–2.3]	1.8 [1.4–2.2]			
Day-7	61	1.8 [1.5–2.3]	1.9 [1.5–2.4]	1.8 [1.4–2.3]			
**ECMO membrane oxygen fraction, %**							
At canulation	90	100 [80–100]	90 [70–100]	100 [92–100]	0.50	0.58	0.05
Day-1	90	90 [70–100]	88 [70–100]	90 [70–100]			
Day-3	80	80 [70–98]	80 [70–90]	80 [60–100]			
Day-7	61	80 [70–100]	80 [70–90]	95 [60–100]			

Data is median [interquartile range].

Data corresponds to patients with per-protocol ventilation on the first day of ECMO run, alive at the given time point. *P* values evaluate the difference between study groups of the variable of interest measured on day-1, day-3 and day-7, using a mixed effects linear regression model, with the interaction term of Group × Time as a fixed effect, and the patient identification code as the random effect to account for the repetition of measurements. In case of a non-significant interaction, the independent association of Group and Time was evaluated. * indicates that there was a significant difference ($P<0.05$) between study groups at this time point if the interaction term was significant. There was no significant difference between study groups during the pre-ECMO period.

ΔP8: ultraprotective strategy in pressure mode with a driving pressure of 8 cmH_2O; ECMO: extracorporeal membrane oxygenation; $PaCO_2$: arterial partial pressure in CO_2; PBW: predicted body weight; VT1: quasi-apneic ventilatory strategy with a tidal volume of 1 $ml.kg^{-1}$ predicted body weight

Supplemental Table 3. Quantitative CT study

Variables	All patients N=32	VT1 N=15	ΔP8 N=17	P value
Delay between ECMO start and CT study, days	1 [0–2]	1 [1–2]	1 [0–1]	0.06
**Ventilator settings and respiratory physiology at time of quantitative CT study**				
Set PEEP, cmH_2O	14 [14–15]	15 [10–15]	14 [14–15]	0.75
Plateau pressure, cmH_2O	21 [19–22]	19 [18–20]	22 [22–22]	<0.01
Tidal volume, $ml.kg^{-1}$ PBW	1 [0.9–1.2]	1 [1–1]	1.2 [0.9–1.8]	0.27
Respiratory rate, bpm	8 [5–14]	5 [5–7]	14 [10–15]	<0.01
PaO_2/FiO_2, mmHg	84 [68–93]	72 [64–88]	88 [77–102]	0.054
**Quantitative CT variables**				
Non inflated lung mass at PEEP 5 cmH_2O, g	1531 [1058–1925]	1393 [994–2027]	1665 [1068–1872]	0.77
End expiratory lung volume at PEEP 5 cmH_2O, ml	239 [137–496]	333 [146–614]	228 [141–402]	0.60
CT-measured tidal volume, $ml.kg^{-1}$ PBW	0.8 [0.7–1]	0.8 [0.7–0.8]	0.8 [0.7–1.5]	0.47
Tidal hyperinflation, $ml.kg^{-1}$ PBW	0.03 [0.01–0.11]	0.02 [0.01–0.13]	0.04 [0.01–0.1]	0.64
Lung recruitment between PEEP 5 and 15 cmH_2O, percentage of total lung mass	5 [3–8]	5 [4–10]	4 [3–7]	0.20

Data is median [interquartile range].
CT: computerized tomography; ΔP8: ultraprotective strategy in pressure mode with a driving pressure of 8 cmH_2O; FiO_2: inspired fraction in O_2; PaO_2: arterial partial pressure in O_2; PBW: predicted body weight; PEEP: positive end-expiratory pressure; VT1: quasi-apneic ventilatory strategy with a tidal volume of 1 $ml.kg^{-1}$ predicted body weight;

Supplemental Table 4. Univariate and multivariate analyses of variables associated with ECMO successful weaning

Variables	Univariate sHR [95% confidence interval)	Univariate P value	Multivariate sHR [95% confidence interval)	Multivariate P value
Age, per 1 year increase	0.99 [0.97–1.01]	0.44	§§	
Male sex, female is the reference	0.46 [0.27–0.78]	<0.01	0.54 [0.31–0.93]	0.03
Body mass index, per 1 $kg.m^{-2}$ increase	0.98 [0.95–1.01]	0.11	§§	
ARDS risk factor is viral pneumonitis (reference is any other risk factor)	0.43 [0.26–0.73]	<0.01	0.30 [0.15–0.61]	<0.01
SAPS-2 score at ICU admission*, per 1 point increase	1.00 [0.98–1.01]	0.59	§§	
Non respiratory SOFA score prior to ECMO**, per 1 point increase	0.98 [0.92–1.04]	0.52	0.92 [0.85–0.99]	0.02
ECMO indication was refractory hypoxemia (reference is hypercapnia)	0.82 [0.40–1.66]	0.57	§§	
PaO_2/FiO_2 ratio prior to ECMO, per 1 mmHg increase	1.00 [0.99–1.01]	0.57	§§	
Plateau pressure prior to ECMO, per 1 cmH_2O increase	1.04 [0.99–1.09]	0.13	||	
Normalized respiratory system elastance prior to ECMO, per 1 $cmH_2O.ml^{-1}.kg^{-1}$ PBW	1.17 [0.92–1.48]	0.20	§§	
Fluid balance on ECMO day, per 1 L increase	1.06 [1.01–1.12]	0.03	1.06 [1.02–1.11]	0.01
Delay between intubation and ECMO, per 1 day increase	0.94 [0.88–1.01]	0.10	§§	
After period (ΔP8 strategy, reference is the before period [VT1 strategy])	1.08 [0.64–1.82]	0.77	0.99 [0.53–1.84]	0.98
Delay between study first inclusion and patient inclusion, per 1 day increase	1.00 [1.00–1.00]	0.56	§§	

Univariate and multivariate regression were performed using Fine and Gray competitive risk regression, with the outcome « weaned from ECMO » as the outcome of interest, and death as the competitive risk. Variables inserted in the multivariate model were those physiologically deemed related to the outcome of interest and the

competitive risk (death). Outcomes were censored at day 90. The multivariate model was evaluated on 10 imputed datasets due to missing values of PaO_2/FiO_2 ratio, normalized elastance, plateau pressure and SOFA scores. The model was simplified using backward stepwise selection, with nested models compared together using the logLik of models applied to imputed datasets. Interactions were systematically checked for. The final model's distribution of Schoenfeld 's residuals were also checked.
*: SAPS-2 score to which points related to age were removed
**: SOFA score to which points related to respiratory failure were removed
||: not inserted into the multivariable model due to collinearity with normalized elastance of the respiratory system
§§: variable not retained in the final model after stepwise backward selection
ARDS: acute respiratory distress syndrome; ΔP8: ultraprotective strategy in pressure mode with a driving pressure of 8 cmH_2O; ECMO: extracorporeal membrane oxygenation; PBW: predicted body weight; SAPS-2: simplified acute physiology score 2; sHR: subdistribution hazard ratio; SOFA: sepsis-related organ failure assessment; VT1: quasi-apneic ventilatory strategy with a tidal volume of 1 $ml.kg^{-1}$ predicted body weight

Supplemental Table 5. Matched population characteristics at baseline and clinical outcomes

Variables	All patients N=62	VT1 N=31	ΔP8 N=31	P value
Age, years	56 [47–62]	56 [49–61]	54 [42–64]	0.64
Sex, male, N (%)	39 (63%)	19 (61%)	20 (65%)	>0.9
Body weight at ICU admission, kg	89 [78–99]	90 [80–100]	88 [75–98]	0.49
Body weight at time of inclusion, kg	85 [80–101]	89 [83–102]	84 [71–100]	0.22
Fluid balance at time of inclusion, kg	0 [-4–1]	0 [-4–1]	0 [-3–0]	0.66
Body mass index, $kg.m^{-2}$	30 [26–35]	31 [27–35]	30 [25–35]	0.82
Comorbidities				
Diabetes, N (%)	14 (23%)	2 (6%)	12 (39%)	0.01
Chronic heart failure, N (%)	1 (2%)	1 (3%)	0 (0%)	>0.9
Chronic kidney disease, N (%)	1 (2%)	0 (0%)	1 (3%)	>0.9
Cancer, N (%)	1 (2%)	1 (3%)	0 (0%)	>0.9
Hematologic malignancy, N (%)	2 (3%)	0 (0%)	2 (6%)	0.49
Medical admission category, N (%)	62 (100%)	31 (100%)	31 (100%)	>0.9
SAPS-2 score at ICU admission	50 [36–59]	43 [33–58]	51 [45–59]	0.22
Total SOFA score at time of inclusion	9 [5–12]	8 [6–12]	10 [5–12]	0.60
ARDS risk factors				>0.9
Viral pneumonia, N (%)	44 (71%)	22 (71%)	22 (71%)	
Bacterial pneumonia, N (%)	17 (27%)	8 (26%)	9 (29%)	
Aspiration pneumonia, N (%)	0 (0%)	0 (0%)	0 (0%)	
Other, N (%)	1 (2%)	1 (3%)	0 (0%)	
None identified, N (%)	0 (0%)	0 (0%)	0 (0%)	
COVID-19 viral pneumonia, N (%)	43 (69%)	21 (68%)	22 (71%)	>0.9
VV-ECMO principal indication				0.49
Refractory hypercapnia, N (%)	2 (3%)	0 (0%)	2 (6%)	
Refractory hypoxemia, N (%)	59 (95%)	30 (97%)	29 (94%)	
Delay between ICU admission and intubation, days	1 [0–3]	0 [0–2]	1 [0–3]	0.12
Delay between intubation and VV-ECMO, days	4 [2–7]	4 [3–8]	3 [1–6]	0.18
Delay between ICU admission and VV-ECMO, days	6 [4–10]	7 [4–9]	6 [3–10]	0.72
ARDS adjunctive therapies at time of inclusion, before VV-ECMO				
Neuromuscular blockade, N (%)	62 (100%)	31 (100%)	31 (100%)	>0.9
Inhaled nitric oxide, N (%)	42 (68%)	19 (61%)	23 (74%)	0.42
Prone positioning, N (%)	62 (100%)	31 (100%)	31 (100%)	>0.9
Renal replacement therapy, N (%)	11 (18%)	6 (19%)	5 (16%)	>0.9
Norepinephrine administration, N (%)	28 (45%)	13 (42%)	15 (48%)	0.59

Norepinephrine dose (tartrate), µg.min^{-1}.kg^{-1}	0 [0–0.34]	0 [0–0.41]	0.01 [0–0.29]	0.53
Arterial lactate concentration, mmol.L^{-1}	1.9 [1.5–2.4]	2 [1.5–2.8]	1.9 [1.5–2.2]	0.58
Alive at day-90, N (%)	23 (37%)	9 (29%)	14 (45%)	0.29
ECMO-free days at day-90, days	0 [0–78]	0 [0–72]	0 [0–80]	0.38
ECMO and vital status at day-90				0.45
Alive with ECMO, N (%)	2 (3%)	0 (0%)	2 (6%)	
Alive and ECMO-free, N (%)	21 (34%)	9 (29%)	12 (39%)	
Death while under ECMO, N (%)	31 (50%)	17 (55%)	14 (45%)	
Death after ECMO successful weaning, N (%)	8 (13%)	5 (16%)	3 (10%)	
Time to successful ECMO weaning in day-90 survivors, days	9 [6–13]	10 [5–13]	9 [7–12]	0.80
Alive and mechanical ventilation-free at day-90, N (%)	19 (31%)	8 (26%)	11 (35%)	0.50
Ventilator-free days at day-90, days	0 [0–55]	0 [0–0]	0 [0–71]	0.16
ICU length of stay in day-90 survivors, days	26 [21–47]	27 [22–47]	25 [20–49]	0.62
Hospital length of stay in day-90 survivors, days	64 [52–95]	91 [78–124]	52 [37–63]	0.01

Data is median [interquartile range] or count (percentage).

ARDS: acute respiratory distress syndrome; ΔP8: ultraprotective strategy in pressure mode with a driving pressure of 8 cmH_2O; ICU: intensive care unit; SAPS-2: simplified acute physiology score 2; SOFA: sepsis-related organ failure assessment; VT1: quasi-apneic ventilatory strategy with a tidal volume of 1 ml.kg^{-1} predicted body weight; VV-ECMO: veno-venous extracorporeal membrane oxygenation

Supplemental Table 6. Matched vs discarded population characteristics at baseline and outcomes at day 90

Variables	All patients<br>N=121	Discarded<br>N=59	Matched<br>N=62	P value
Age, years	51 [41–61]	46 [35–56]	56 [47–62]	<0.01
Sex, male, N (%)	74 (61%)	35 (59%)	39 (63%)	0.71
Body weight at ICU admission, kg	87 [74–98]	85 [72–96]	89 [78–99]	0.28
Body weight at time of inclusion, kg	85 [72–100]	86 [68–98]	85 [80–101]	0.46
Fluid balance at time of inclusion, kg	0 [-3–1]	0 [-2–0]	0 [-4–1]	0.53
Body mass index, $kg.m^{-2}$	30 [26–34]	30 [26–34]	30 [26–35]	0.73
Comorbidities				
Diabetes, N (%)	25 (21%)	11 (19%)	14 (23%)	0.66
Chronic heart failure, N (%)	1 (1%)	0 (0%)	1 (2%)	>0.9
Chronic kidney disease, N (%)	1 (1%)	0 (0%)	1 (2%)	>0.9
Cancer, N (%)	1 (1%)	0 (0%)	1 (2%)	>0.9
Hematologic malignancy, N (%)	8 (7%)	6 (10%)	2 (3%)	0.16
Medical admission category, N (%)	114 (94%)	52 (88%)	62 (100%)	0.01
SAPS-2 score at ICU admission	52 [36–61]	53 [36–61]	50 [36–59]	0.63
Total SOFA score at time of inclusion	9 [7–13]	8 [8–13]	9 [5–12]	0.65
ARDS risk factors				0.17
Viral pneumonia, N (%)	78 (64%)	34 (58%)	44 (71%)	
Bacterial pneumonia, N (%)	33 (27%)	16 (27%)	17 (27%)	
Aspiration pneumonia, N (%)	3 (2%)	3 (5%)	0 (0%)	
Other, N (%)	5 (3%)	4 (5%)	1 (2%)	
None identified, N (%)	1 (1%)	1 (2%)	0 (0%)	
COVID-19 viral pneumonia, N (%)	73 (60%)	30 (51%)	43 (69%)	0.04
VV-ECMO principal indication				<0.01
Refractory hypercapnia, N (%)	18 (15%)	16 (27%)	2 (3%)	
Refractory hypoxemia, N (%)	102 (84%)	43 (73%)	59 (95%)	
Delay between ICU admission and intubation, days	1 [0–2]	1 [0–1]	1 [0–3]	0.27
Delay between intubation and VV-ECMO, days	3 [1–7]	3 [1–6]	4 [2–7]	0.33
Delay between ICU admission and VV-ECMO, days	5 [2–10]	4 [1–10]	6 [4–10]	0.19
ARDS adjunctive therapies at time of inclusion, before VV-ECMO				
Neuromuscular blockade, N (%)	120 (99%)	58 (98%)	62 (100%)	>0.9
Inhaled nitric oxide, N (%)	76 (63%)	34 (58%)	42 (68%)	0.45
Prone positioning, N (%)	120 (99%)	58 (98%)	62 (100%)	0.49
Renal replacement therapy, N (%)	19 (16%)	8 (14%)	11 (18%)	0.62
Norepinephrine administration, N (%)	63 (52%)	35 (59%)	28 (45%)	0.25
Norepinephrine dose (tartrate), $\mu g.min^{-1}.kg^{-1}$	0.03 [0–0.49]	0.14 [0–0.58]	0 [0–0.34]	0.16

Arterial lactate concentration, mmol.L^{-1}	1.9 [1.5–3]	1.9 [1.4–3.1]	1.9 [1.5–2.4]	0.93
Alive at day-90, N (%)	43 (36%)	20 (34%)	23 (37%)	0.85
ECMO-free days at day-90, days	0 [0–77]	0 [0–74]	0 [0–78]	0.94
ECMO and vital status at day-90				0.77
Alive with ECMO, N (%)	2 (2%)	0 (0%)	2 (3%)	
Alive and ECMO-free, N (%)	41 (34%)	20 (34%)	21 (34%)	
Death while under ECMO, N (%)	62 (51%)	31 (53%)	31 (50%)	
Death after ECMO successful weaning, N (%)	16 (13%)	8 (14%)	8 (13%)	
Time to successful ECMO weaning in day-90 survivors, days	7 [4–13]	6 [4–14]	9 [6–13]	0.40
Alive and invasive mechanical ventilation-free at day-90, N (%)	35 (29%)	16 (27%)	19 (31%)	0.50
Ventilator-free days at day-90, days	0 [0–41]	0 [0–20]	0 [0–55]	0.55
ICU length of stay in day-90 survivors, days	27 [21–58]	32 [18–61]	26 [21–47]	0.76
Hospital length of stay in day-90 survivors, days	58 [43–97]	48 [39–97]	64 [52–95]	0.27

Data is median [interquartile range] or count (percentage).
ARDS: acute respiratory distress syndrome; ΔP8: ultraprotective strategy in pressure mode with a driving pressure of 8 cmH_2O; ICU: intensive care unit; SAPS-2: simplified acute physiology score 2; SOFA: sepsis-related organ failure assessment; VT1: quasi-apneic ventilatory strategy with a tidal volume of 1 ml.kg^{-1} predicted body weight; VV-ECMO: veno-venous extracorporeal membrane oxygenation

Supplementary figures

Supplemental Figure 1. Study flowchart

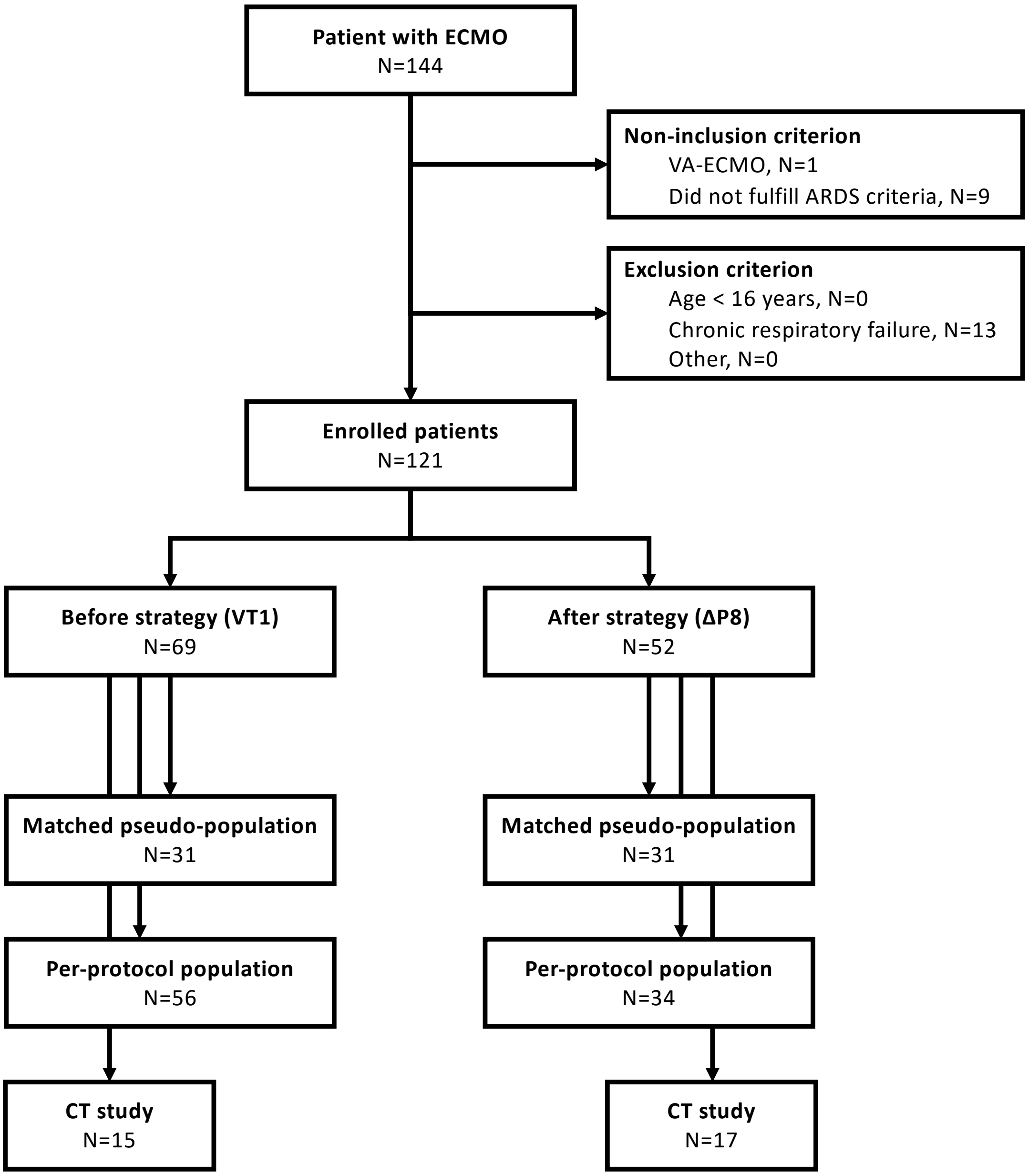

ARDS: acute respiratory distress syndrome; ΔP8: ultraprotective strategy in pressure mode with a driving pressure of 8 cmH_2O; VA-ECMO: veno-arterial extracorporeal membrane oxygenation; VT1: quasi-apneic ventilatory strategy with a tidal volume of 1 $ml.kg^{-1}$ predicted body weight; VV-ECMO: veno-venous extracorporeal membrane oxygenation ; CT : computed tomography

Supplemental Figure 2. Rate of use of neuromuscular blockade and prone positioning in all patients over the first 7 days of VV-ECMO

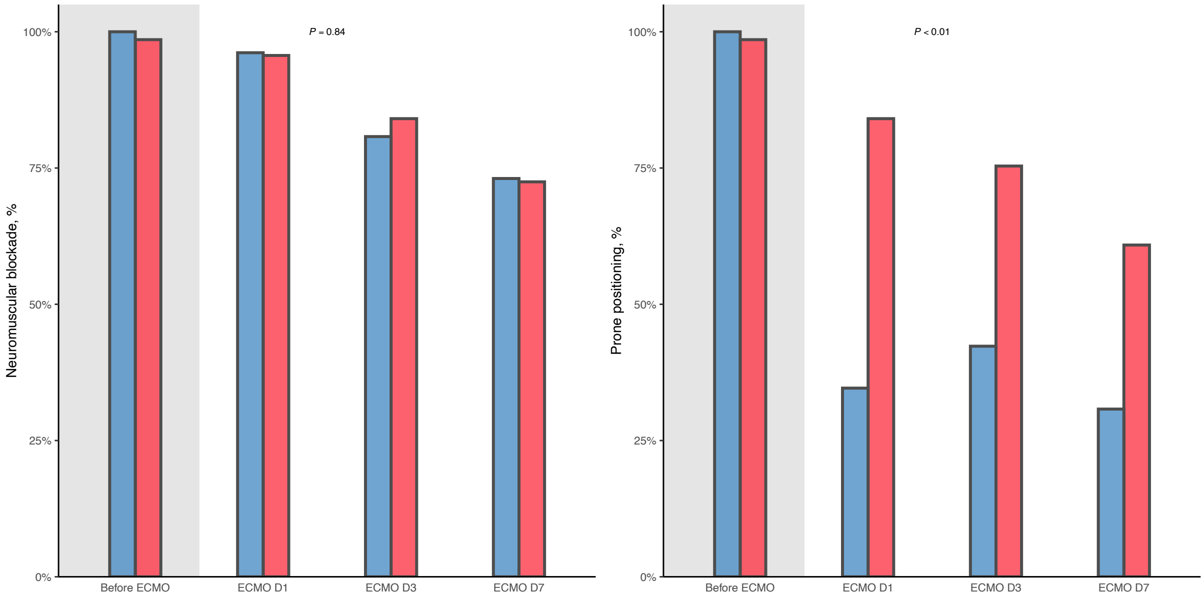

The figure shows the percentage of use of neuromuscular blockade (A) and prone positioning (B) during the first 7 days of follow-up after ECMO canulation, in each study group (VT1 strategy in red, ΔP8 strategy in blue).

ΔP8: ultraprotective strategy in pressure mode with a driving pressure of 8 cmH_2O; VT1: quasi-apneic ventilatory strategy with a tidal volume of 1 $ml.kg^{-1}$ predicted body weight; VV-ECMO: veno-venous extracorporeal membrane oxygenation

Supplemental Figure 3. Ventilatory mode used over time in all patients over the first 7 days of VV-ECMO

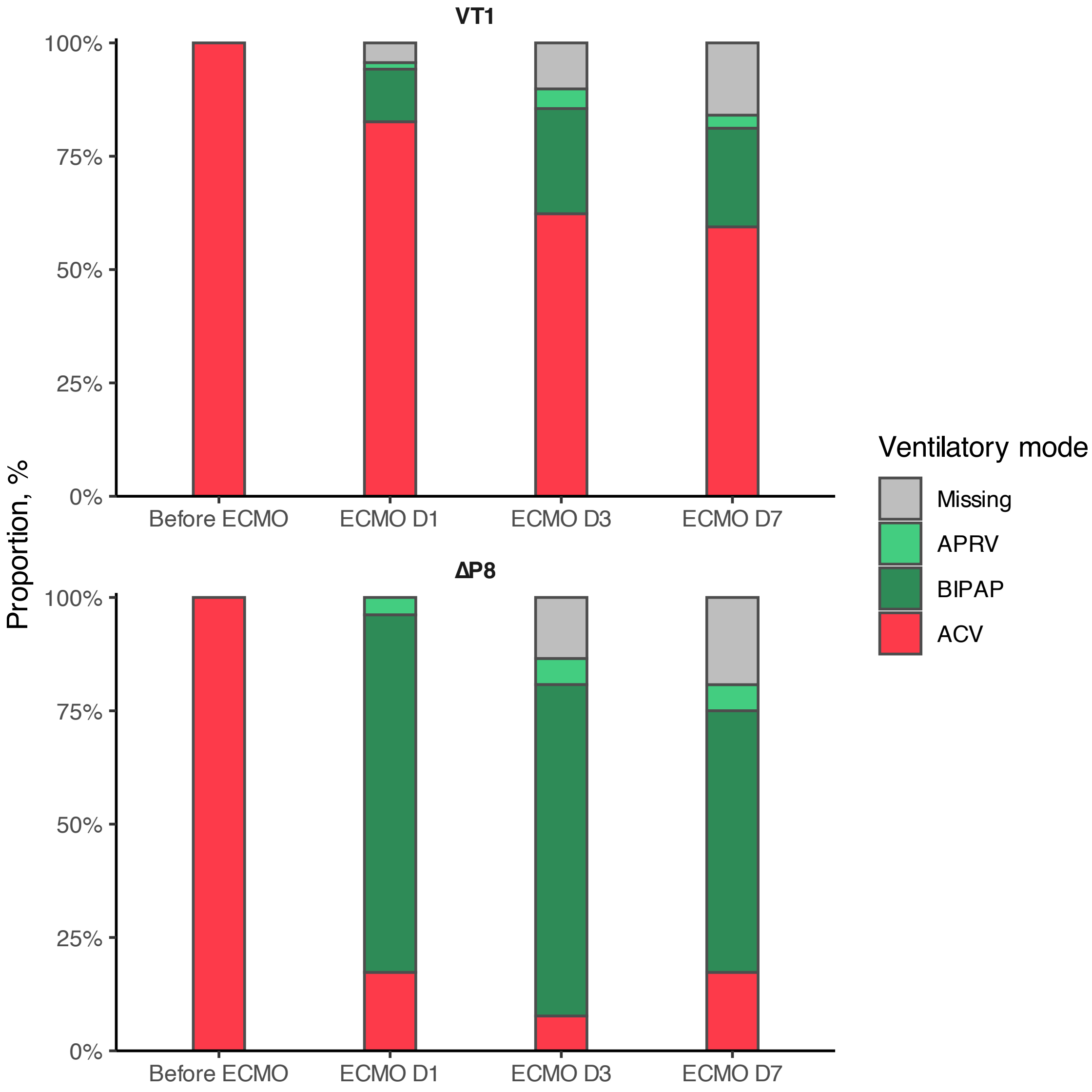

The figure shows the percentage of use of controlled ventilatory modes (APRV, BIPAP or ACV) during the first 7 days of follow-up after ECMO canulation, in each study group (VT1 strategy in red, ΔP8 strategy in blue).

ΔP8: ultraprotective strategy in pressure mode with a driving pressure of 8 cmH_2O; ACV: assit-controlled volume; APRV: airway pressure release ventilation; BIPAP: biphasic intermittent positive airway pressure; VT1: quasi-apneic ventilatory strategy with a tidal volume of 1 $ml.kg^{-1}$ predicted body weight; VV-ECMO: veno-venous extracorporeal membrane oxygenation

Supplemental Figure 4. Application of ventilatory strategies in all patients on day 1 of VV-ECMO

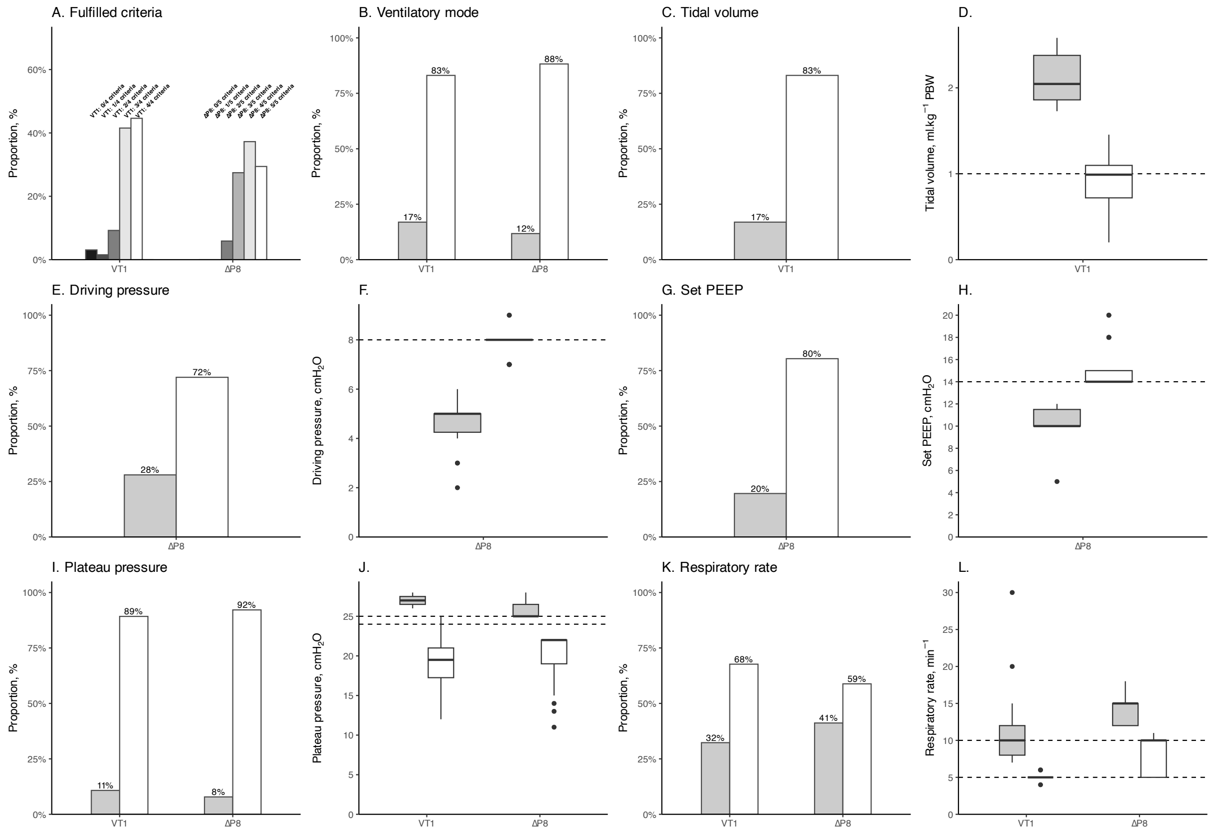

The figure shows the number of criteria effectively applied for each ventilatory strategy (panel A) and the rate of per-protocol ventilatory mode (volume or pressure, as per protocol in white, protocol violation in grey) used in each group (B) on the first day of ECMO canulation. From panel C to L, the panels show the percentage of patients effectively receiving (in white) or not (in grey) the ventilatory setting or objective they were assigned to as was defined by the protocol. Next to each barplot is represented the observed values of the parameter of interest, based on the protocol application or violation. The leading reason for protocol violation was an increase in respiratory rate with both strategies (panels K and L). In the ΔP8 strategy, PEEP setting and driving pressure setting were not respected in a quarter of cases with the application of lower levels of both pressures.

ΔP8: ultraprotective strategy in pressure mode with a driving pressure of 8 cmH_2O; PBW: predicted body weight; PEEP: positive end-expiratory pressure; VT1: quasi-apneic ventilatory strategy with a tidal volume of 1 ml.kg^{-1} predicted body weight; VV-ECMO: veno-venous extracorporeal membrane oxygenation.

Supplemental Figure 5. Respiratory mechanics over the first 7 days after VV-ECMO canulation in patients receiving per-protocol ventilation

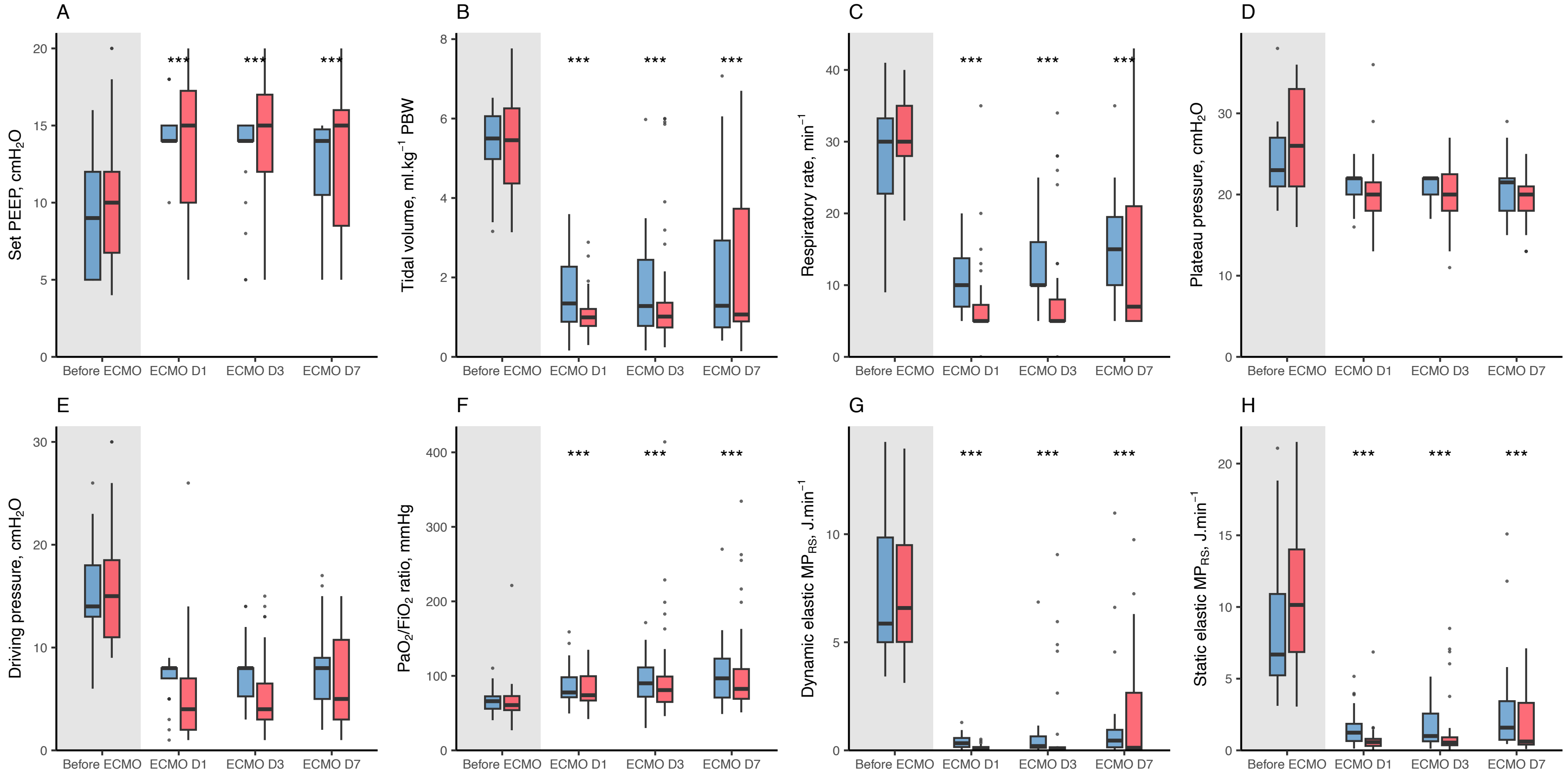

The figure shows the course over time of set PEEP (A), tidal volume (B), respiratory rate (C), plateau pressure (D), driving pressure (E), PaO_2/FiO_2 ratio (F), dynamic elastic mechanical power (G), static elastic mechanical power (H) in the patients receiving the VT1 strategy (in red) or the ΔP8 strategy (in blue) in the subpopulation with per-protocol ventilation application on the first day of ECMO run. Per-protocol ventilation defined as the fulfilment of at least 3 out of 4 of the protocol's criteria for the VT1 strategy (N=56 out of 69), and 4 out of 5 criteria for the ΔP8 strategy (N=34 out of 52). Data is represented by mean of boxplots (with median and first and third quartile) and outliers (black dots). For each studied variable, the difference between groups was evaluated using mixed effect regression models applied to measurements obtained on day-1, day-3 and day-7, with the interaction of Time × Group as the independent variable, and the patient identification code as the random intercept. In case of a significant interaction, a post-hoc pairwise comparison was performed between study groups, adjusted using the Sidak method. In case of a non-significant interaction term but with a significant effect of Group, the results of pairwise comparisons were given, using the Sidak method. The was no significant differences in baseline values of displayed variables. The *** in panels A, B, C, F, G and H indicate a significant difference at this time point between study groups ($P<0.05$).
ΔP8: ultraprotective strategy in pressure mode with a driving pressure of 8 cmH_2O; MP_{RS}: respiratory system mechanical power, PaO_2/FiO_2: ratio of arterial oxygen partial pressure to the fraction of inspired oxygen; PBW: predicted body weight; PEEP: positive end-expiratory pressure; VT1: quasi-apneic ventilatory strategy with a tidal volume of 1 $ml.kg^{-1}$ predicted body weight; VV-ECMO: veno-venous extracorporeal membrane oxygenation.

Supplemental Figure 6. Respiratory mechanics on the first day of ECMO canulation in all patients, as a function of the normalized elastance before ECMO canulation

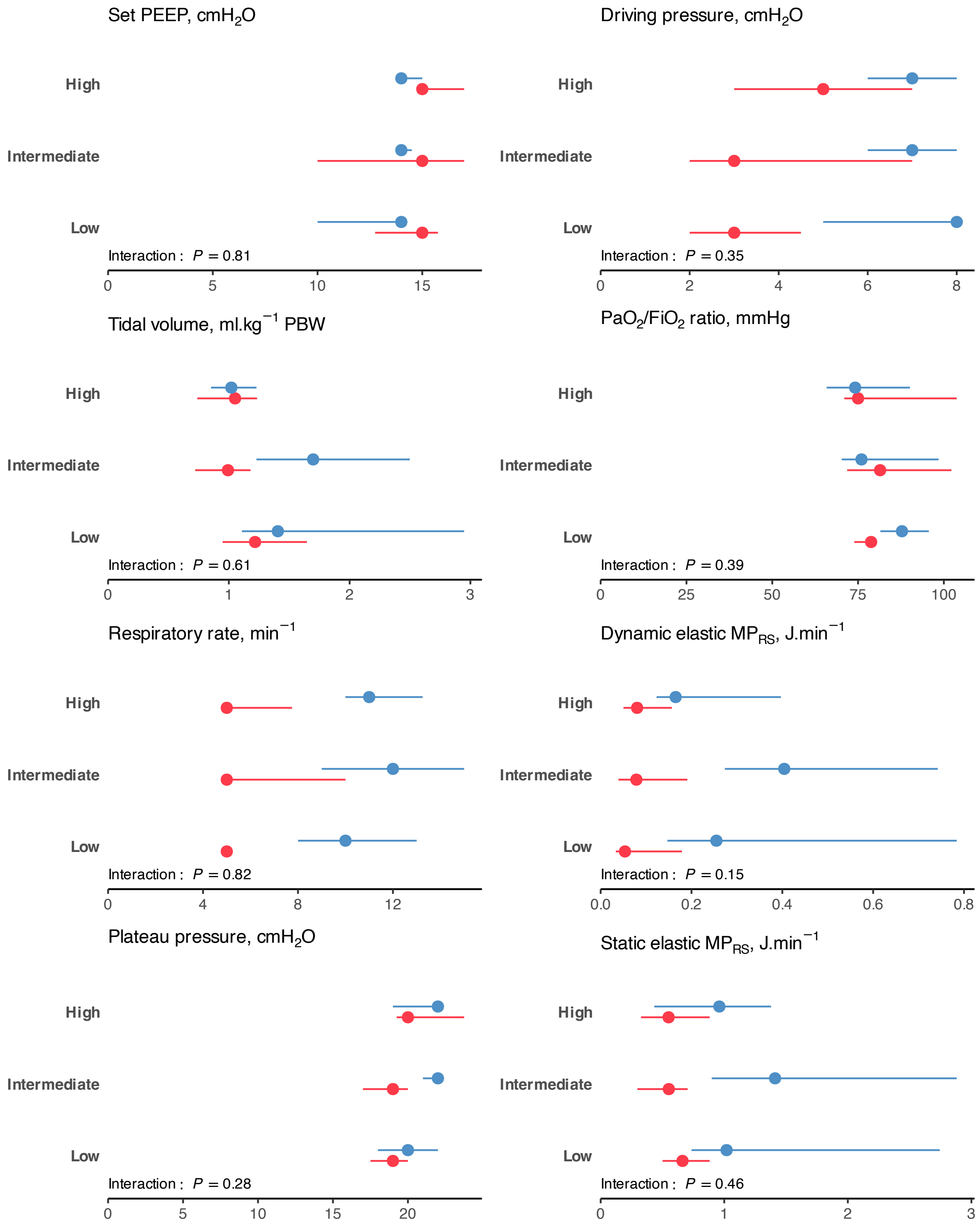

The figure shows the values of set PEEP, tidal volume, respiratory rate, plateau pressure, driving pressure, PaO_2/FiO_2 ratio, dynamic elastic mechanical power, static elastic mechanical power in the

patients receiving the VT1 strategy (in red) or the ΔP8 strategy (in blue), as a function of their normalized elastance observed prior to VV-ECMO canulation. Normalized elastance was classified as low (≤ 2 $cmH_2O.ml^{-1}.kg^{-1}$ PBW, N=17), intermediate (between 2 and 3 $cmH_2O.ml^{-1}.kg^{-1}$ PBW, N=44) or high (>3 $cmH_2O.ml^{-1}.kg^{-1}$ PBW, N=35) (13). Baseline normalized elastance was missing in 25 patients prior to ECMO (in relation with missing plateau pressure measurements), and these patients were excluded from this analysis. In each panel, the interaction of study group and normalized elastance class was evaluated by mean of a linear regression model. The *P* value for interaction is indicated in each panel.

ΔP8: ultraprotective strategy in pressure mode with a driving pressure of 8 cmH_2O; MP_{RS}: respiratory system mechanical power, PaO_2/FiO_2: ratio of arterial oxygen partial pressure to the fraction of inspired oxygen; PBW: predicted body weight; PEEP: positive end-expiratory pressure; VT1: quasi-apneic ventilatory strategy with a tidal volume of 1 $ml.kg^{-1}$ predicted body weight; VV-ECMO: veno-venous extracorporeal membrane oxygenation.

Supplemental Figure 7. Effects on normalized elastance and mechanical power of increasing tidal volumes and respiratory rates

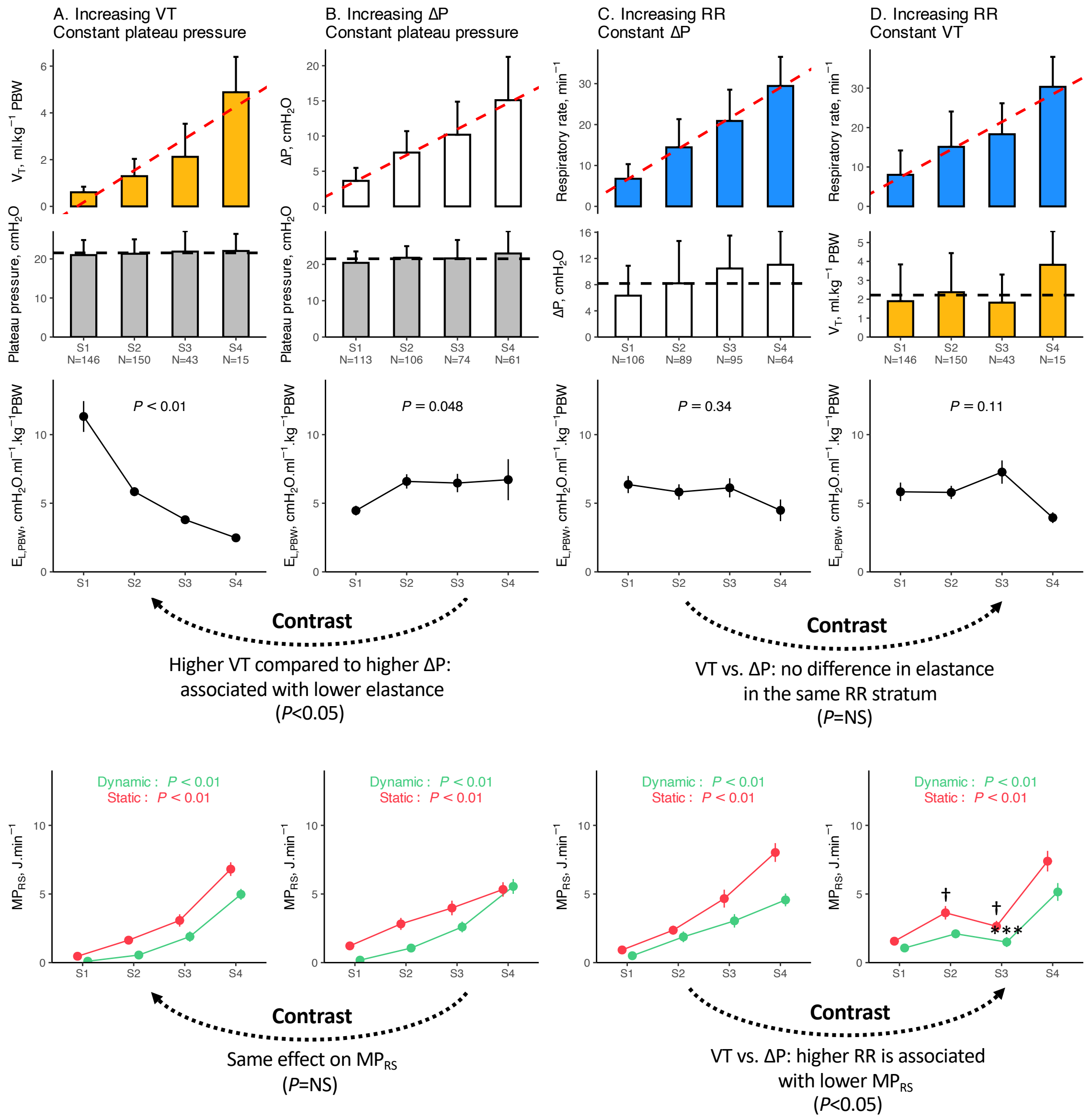

Using double stratification (obtaining subgroups of observations with matched mean levels for one variable but different mean levels of another ranking variable), the figure shows the association of increasing tidal volume (column A) and driving pressure (column B, same analysis as presented in column C of figure 3 of the main manuscript), in observations with similar levels of plateau pressure, with normalized elastance and mechanical power. Similarly, columns C and D show the association in increasing respiratory rates in observations with similar levels of driving pressure (column C) or tidal volume (column D) with the same parameters. The first row depicts the effect size of the variable of interest (mean value ± standard deviation) found in each stratum, along with the value of the matched variable in the second row (plateau pressure, driving pressure, or tidal volume), and the number of observations in each stratum. The red dotted line corresponds to the slope of increasing VT, driving pressure or respiratory rate, respectively. The black dotted lines correspond to the mean value of the

other ranking variable (plateau pressure, ΔP or tidal volume). The third row shows the mean normalized elastance (± standard error) for each stratum. The fourth row shows the means values (± standard error) of static elastic (red points and lines) and dynamic elastic mechanical power (green points and lines). *P* values examine the association of normalized elastance on the one side, and mechanical power on the other, between strata (as a categorical variable), using linear regression. Note that in panels C and D, subsampling did not allow homogenous distribution of mean ΔP or VT in each stratum (especially in the S4 subsample). Also, note that normalized elastance and mechanical power are mathematically related to driving pressure and VT. The dotted arrows indicate the contrasts between subsamples A and B, and D and D, within each stratum, using pairwise comparison adjusted for multiple comparison.

***: significant difference in dynamic elastic mechanical power in this stratum in pairwise comparison between C and D.

†: significant difference in static elastic mechanical power in this stratum in pairwise comparison between C and C.

ΔP: driving pressure; $E_{L,PBW}$: normalized elastance of the respiratory system; MP_{RS}: respiratory system mechanical power; PBW: predicted body weight; PEEP: positive end-expiratory pressure; RR: respiratory rate; V_T: tidal volume

Supplemental Figure 8. Time to successful ECMO weaning in matched patients alive at day 90

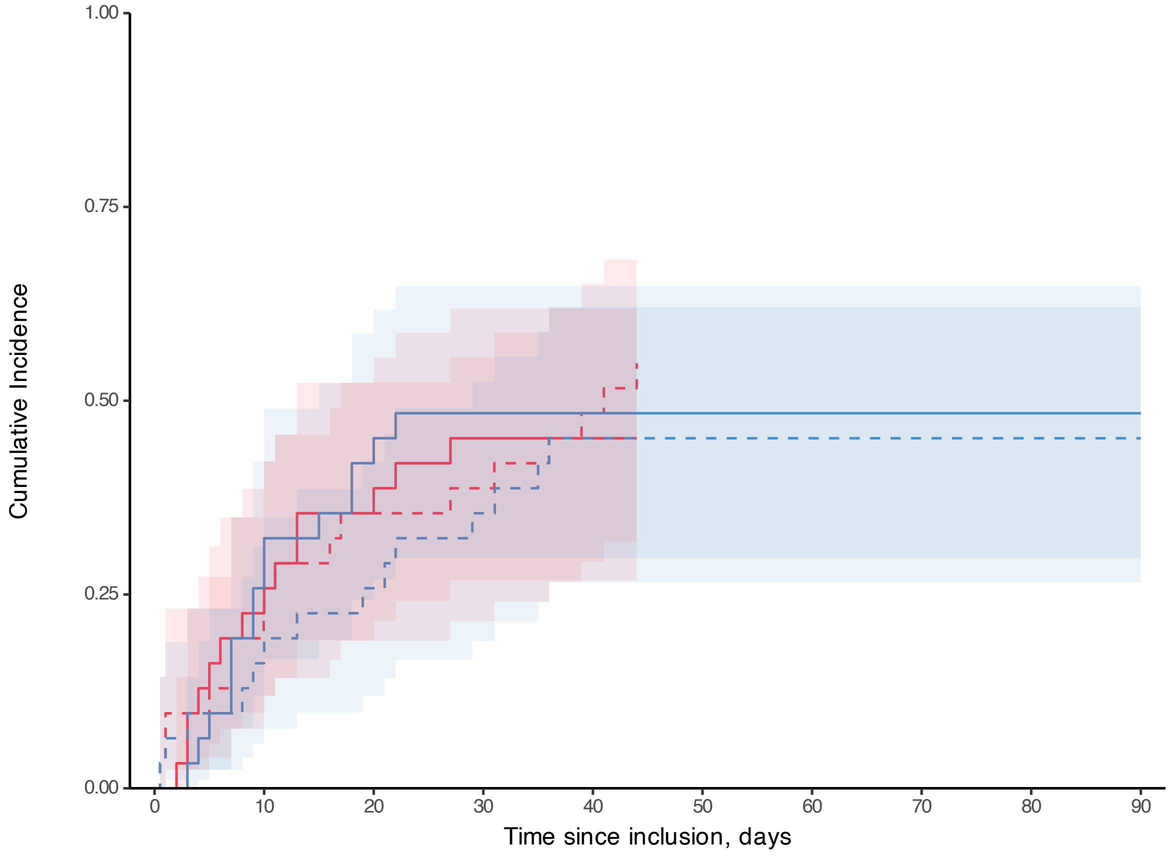

VT1										
At Risk	31	17	9	5	2	0	0	0	0	0
Events	0	16	23	26	29	31	31	31	31	31
ΔP8										
At Risk	31	18	10	5	2	2	2	2	2	2
Events	0	16	22	26	29	29	29	29	29	29

The figure shows the time to successful VV-ECMO weaning in the matched cohort (broad lines), in patients of the before period (VT1 strategy, in red), and the after period (ΔP8 strategy, in blue). The represented cumulative incidence accounts for the competitive risk of death in the matched cohort (dashed lines). Patients alive and with VV-ECMO at day 90 were censored at this time. The red and blue shades depict the 95% interval of the cumulative incidence at a given time. The number of patients at-risk and with events are given below the figure. The red lines stop after day 50 because all patients in this group had met one or the other competitive outcomes (ECMO weaning or death). No significant difference between study groups on the time to successful ECMO weaning was observed in this analysis (*P*=0.85).

ΔP8: ultraprotective strategy in pressure mode with a driving pressure of 8 cmH_2O; VT1: quasi-apneic ventilatory strategy with a tidal volume of 1 $ml.kg^{-1}$ predicted body weight; VV-ECMO: veno-venous extracorporeal membrane oxygenation

Supplemental References

1. ARDS Definition Task Force, Ranieri VM, Rubenfeld GD, Thompson BT, Ferguson ND, Caldwell E, et al. Acute respiratory distress syndrome: the Berlin Definition. JAMA. 2012;307:2526–33.

2. Le Gall JR, Lemeshow S, Saulnier F. A new Simplified Acute Physiology Score (SAPS II) based on a European/North American multicenter study. JAMA. 1993;270:2957–63.

3. Vincent JL, Moreno R, Takala J, Willatts S, De Mendonça A, Bruining H, et al. The SOFA (Sepsis-related Organ Failure Assessment) score to describe organ dysfunction/failure. On behalf of the Working Group on Sepsis-Related Problems of the European Society of Intensive Care Medicine. Intensive Care Med. 1996;22:707–10.

4. Ventilation with Lower Tidal Volumes as Compared with Traditional Tidal Volumes for Acute Lung Injury and the Acute Respiratory Distress Syndrome. The New England Journal of Medicine. 2000;

5. Gattinoni L, Tonetti T, Cressoni M, Cadringher P, Herrmann P, Moerer O, et al. Ventilator-related causes of lung injury: the mechanical power. Intensive Care Med. 2016;42:1567–75.

6. Giosa L, Busana M, Pasticci I, Bonifazi M, Macrì MM, Romitti F, et al. Mechanical power at a glance: a simple surrogate for volume-controlled ventilation. Intensive Care Med Exp. 2019;7:61.

7. Schmidt M, Hajage D, Lebreton G, Dres M, Guervilly C, Richard JC, et al. Prone Positioning During Extracorporeal Membrane Oxygenation in Patients With Severe ARDS: The PRONECMO Randomized Clinical Trial. JAMA. 2023;330:2343–53.

8. Guérin C, Reignier J, Richard J-C, Beuret P, Gacouin A, Boulain T, et al. Prone Positioning in Severe Acute Respiratory Distress Syndrome. N Engl J Med. 2013;368:2159–68.

9. Sessler CN, Gosnell MS, Grap MJ, Brophy GM, O'Neal PV, Keane KA, et al. The Richmond Agitation-Sedation Scale: validity and reliability in adult intensive care unit patients. Am J Respir Crit Care Med. 2002;166:1338–44.

10. Papazian L, Perrin G, Seghboyan J-M, Guérin C. Neuromuscular Blockers in Early Acute Respiratory Distress Syndrome. n engl j med. 2010;

11. Meduri GU, Golden E, Freire AX, Taylor E, Zaman M, Carson SJ, et al. Methylprednisolone infusion in early severe ARDS: results of a randomized controlled trial. Chest. 2007;131:954–63.

12. Penarrubia L, Verstraete A, Orkisz M, Davila E, Boussel L, Yonis H, et al. Precision of CT-derived alveolar recruitment assessed by human observers and a machine learning algorithm in moderate and severe ARDS. Intensive Care Med Exp. 2023;11:8.

13. Amato MB, Meade MO, Slutsky AS, Brochard L, Costa EL, Schoenfeld DA, Stewart TE, Briel M, Talmor D, Mercat A, Richard JC, Carvalho CR, Brower RG. Driving pressure and survival in the acute respiratory distress syndrome. N Engl J Med. 2015;372(8):747-55.
